# VINNA for neonates: Orientation independence through latent augmentations

**DOI:** 10.1162/imag_a_00180

**Published:** 2024-05-30

**Authors:** Leonie Henschel, David Kügler, Lilla Zöllei, Martin Reuter

**Affiliations:** German Center for Neurodegenerative Diseases (DZNE), Bonn, Germany; A.A. Martinos Center for Biomedical Imaging, Massachusetts General Hospital, Boston, MA, USA; Department of Radiology, Harvard Medical School, Boston, MA, USA

**Keywords:** computational neuroimaging, deep learning, structural MRI, artificial intelligence, high-resolution, newborn brain

## Abstract

A robust, fast, and accurate segmentation of neonatal brain images is highly desired to better understand and detect changes during development and disease, specifically considering the rise in imaging studies for this cohort. Yet, the limited availability of ground truth datasets, lack of standardized acquisition protocols, and wide variations of head positioning in the scanner pose challenges for method development. A few automated image analysis pipelines exist for newborn brain Magnetic Resonance Image (MRI) segmentation, but they often rely on time-consuming non-linear spatial registration procedures and require resampling to a common resolution, subject to loss of information due to interpolation and down-sampling. Without registration and image resampling, variations with respect to head positions and voxel resolutions have to be addressed differently. In deep learning, external augmentations such as rotation, translation, and scaling are traditionally used to artificially expand the representation of spatial variability, which subsequently increases both the training dataset size and robustness. However, these transformations in the image space still require resampling, reducing accuracy specifically in the context of label interpolation. We recently introduced the concept of resolution-independence with the Voxel-size Independent Neural Network framework, VINN. Here, we extend this concept by additionally shifting all rigid-transforms into the network architecture with a four degree of freedom (4-DOF) transform module, enabling resolution-aware internal augmentations (VINNA) for deep learning. In this work, we show that VINNA (i) significantly outperforms state-of-the-art external augmentation approaches, (ii) effectively addresses the head variations present specifically in newborn datasets, and (iii) retains high segmentation accuracy across a range of resolutions (0.5–1.0 mm). Furthermore, the 4-DOF transform module together with internal augmentations is a powerful, general approach to implement spatial augmentation without requiring image or label interpolation. The specific network application to newborns will be made publicly available as VINNA4neonates.

## 1 Introduction

Collections of neonatal brain Magnetic Resonance Images (MRIs) are indispensable to understand brain development and to detect early signs of potential developmental disorders. One of the key tasks in MRI analysis is automated segmentation, the labeling of anatomical regions of interest (ROIs) that can be used for quantitative modeling of healthy development, for analyses in population studies, for understanding disease effects as well as a starting point for further neuroimaging tasks. The segmentation of infant MRIs is a challenging and non-trivial undertaking due to the rapid non-linear changes during the postnatal brain growth period, elevated levels of head motion, limited availability of congruent datasets, varying intensity profiles across scanners, protocols and modalities, as well as the inversion of gray-white contrast around the age of 5–9 months (Ajayi-Obe et al., 2000; Dubois et al., 2014; Gilmore et al., 2012; Prastawa et al., 2005). In this paper, we focus on a sub-group of the infant population—newborns—and present a four-degree of freedom (4-DOF) transform module to address two core challenges within this cohort: non-uniform image resolutions (scaling) and increased variability of head positions (rigid transformations = rotation and translation) during image acquisition. The 4-DOF transform module is directly integrated into the network architecture and addresses the variability of head positions internally. As such, it expands and generalizes the distinguishing feature, resolution independence, of the recently published Voxel-size Independent Neural Network (VINN) (Henschel et al., 2022) by rotation and translation transformations. We refer to our new framework as VINN with “internal augmentations” (VINNA).

In contrast to adults, newborn head positions in the scanner are far more diverse due to the scanning conditions (asleep) and overall smaller anatomy. While padding is often used to stabilize the child’s head and to occupy the space between head coil and participant (e.g., foam cushions, pads, mats, pillows, or visco-elastic matters) (Copeland et al., 2021), its standardization is difficult. This results in diverse head orientations within the scanner and potentially high variations among imaging studies.

In addition, there is no de-facto standard resolution for newborn imaging. In the case of research protocols, when more time is available, MRIs are often acquired at higher resolutions to address the small size of brain structures and stronger partial volume effects (Dubois et al., 2019, 2021; Liu et al., 2019). However, the range of recorded resolutions across research and clinical studies is relatively large and heterogeneous, ranging from 0.5 mm to 3.0 mm in past and recent research studies (e.g., NIH-PD (Evans, 2006), BCP (Howell et al., 2019), Developing Human Connectome Project (dHCP) (Bastiani et al., 2019; Makropoulos et al., 2018), HBCD (Volkow et al., 2021)). Similarly, resolutions are not standardized across atlases (UNC 0-1-2 Infant atlases (Shi et al., 2011), Imperial Brain Development atlases (Gousias et al., 2012; Kuklisova-Murgasova et al., 2011; Makropoulos et al., 2016; Serag et al., 2012)), that are often used to guide the automated labeling algorithms.

Traditional tools for newborn or infant segmentation predominantly interpolate images to a single chosen resolution and harmonize the spatial head position via atlas registrations (Cherel et al., 2015; Dai et al., 2013; Makropoulos et al., 2018; Prieto et al., 2019; Shi et al., 2010; Zöllei et al., 2020). Resampling of images can, however, result in loss of information, especially in the context of high-resolution label maps. Furthermore, atlas-guided approaches are usually highly dependent on registration accuracy. For newborns, registration is particularly challenging due to lower tissue contrast, specifically in the T1w scans. Errors in the process are hence common and improvement of the registration, for example, with spatio-temporal information, anatomical constraints, and surface models, is an active field of research (Ahmad et al., 2019; Bozek et al., 2018; Dubois et al., 2021; Garcia et al., 2018; Kuklisova-Murgasova et al., 2011; Lebenberg et al., 2018; Makropoulos et al., 2014; Shi et al., 2010).

The explicit definition of spatial and intensity features can be avoided by using Convolutional Neural Networks (CNNs). In fact, fast deep-learning methods for semantic segmentation are becoming increasingly popular for infant segmentation (Bui et al., 2019; Dolz et al., 2020; Kumar et al., 2018; Moeskops et al., 2015; Nie et al., 2016; Qamar et al., 2020; Wang et al., 2022, 2023; Zeng & Zheng, 2018; Zeng et al., 2023; Zhang et al., 2015). Applicability of deep-learning approaches, however, is generally restricted to domains where sufficiently large training datasets exist. While there have been several initiatives to collect larger neuroimaging cohorts of newborns and infants in recent years (Bastiani et al., 2019; Howell et al., 2019; Makropoulos et al., 2018; Volkow et al., 2021), their size is still relatively small compared to equivalent cohorts in the adult population. Additionally, accompanying manual labels are sparse, due to high annotation costs (time and money) and non-uniform labeling protocols, limiting the pool for supervised training options further. Considering the newborn cohort, the variability in resolution and head positioning is likely underrepresented in the publicly available datasets, questioning whether a network trained on the available pairs of scans and labels can be robust enough without additional augmentation.

**Fig. 1.**
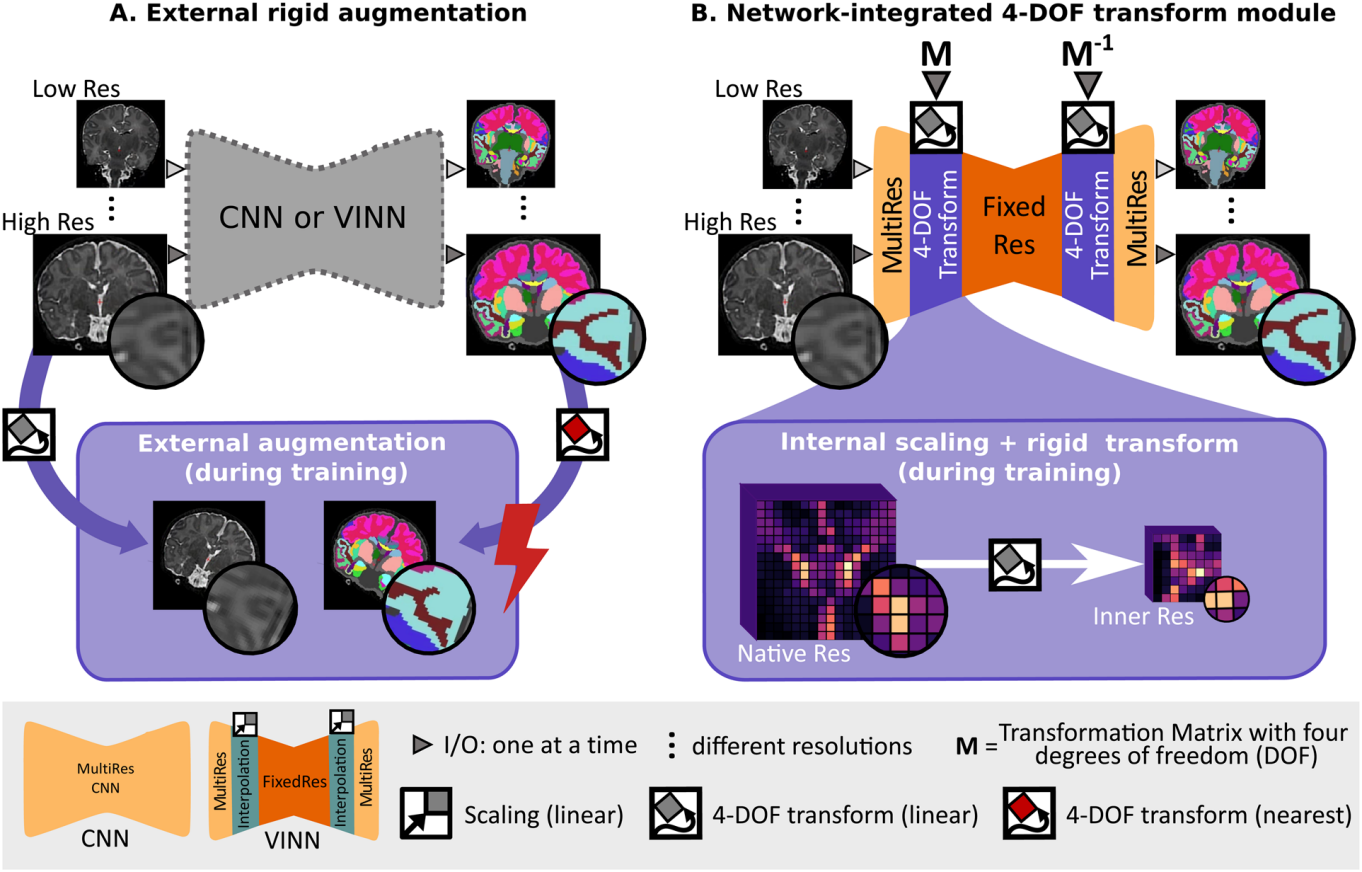
Spatial augmentations in deep-learning networks: (A) One single resolution-ignorant CNN or resolution-independent voxel size independent network (VINN) can learn to segment multiple resolutions and head positions by training on a diverse dataset. External scale, rotation, and translation augmentation (+ external augmentation, A. bottom) diversifies the existing training samples by resampling the images and the reference segmentation maps. Here, however, lossy interpolation and resulting artefacts, especially from nearest-neighbor interpolation of discrete label maps, may result in a loss of structural details and sub-optimal performance. (B) Our 4-DOF transform module (VINNA) completely avoids interpolation of the images and discrete labels by integrating the interpolation step into the network architecture itself (B. bottom). Rotations, translations, and scalings applied in the first interpolation block are later reversed, assuring segmentations to be in the original image orientation. Furthermore, the explicit transition from the native resolution to a normalized internal resolution facilitates an understanding of the difference between image features (MultiRes blocks with distances measured in voxels) and anatomical features (FixedRes inner blocks with normalized distances).

The most widely used solution to artificially increase the training set size, robustness, and generalizability of deep-learning methods has been traditional data augmentation, such as rotation, scaling, or translation (Fig. 1A). In this case, both images and their labelmaps are interpolated to a new random position during training. Interpolation, however, in the native image space requires resampling of the discrete ground truth segmentations, resulting in information loss (e.g., from lossy nearest-neighbor (NN) interpolation) and reduction in accuracy (Henschel et al., 2022).

With the VINN architecture (Henschel et al., 2022), we recently established the first network for resolution-independent deep learning, which effectively circumvents scaling augmentation and subsequent external resampling, while leveraging information across datasets of varying resolutions. In VINN, the classic fixed-factor integer down- and up-scale transitions, often implemented via pooling operations in UNet-like architectures (Ronneberger et al., 2015), are replaced with a flexible re-scaling for the first and last scale transitions. This network-integrated resolution-normalization allows for segmentation in the native space during both, training and inference. In adults, this approach has been shown to outperform fixed-resolution CNNs as well as resolution-ignorant CNNs trained with external scaling augmentation, and to improve performance both for sub-millimeter and one-millimeter scans.

Since newborn datasets are often acquired at various native resolutions and are particularly subject to partial volume effects, the resolution-normalization feature offers a basis to improve segmentation performance here as well. As is, VINN only addresses scaling and would still require external augmentations, and hence label interpolation, to address the increased variability of head positions and limited availability of training data for newborns. With our VINNA and its 4-DOF transform module, we now close this gap and propose to shift away from classical data augmentation towards a detail-preserving internal augmentation scheme (Fig. 1B). While avoiding any type of label interpolation, we extend VINN’s network-integrated resolution-normalization with spatial augmentations (i.e., rotation and translation). At the first layer scale transition, the feature maps are hence not only flexibly rescaled, but also randomly transformed to imitate the position variations commonly found in newborns, subsequently increasing the training distribution.

In conclusion, VINNA and its 4-DOF transform module effectively address the challenges associated with newborn segmentation, namely variation in head positions and resolutions in the context of limited data availability. The four key contributions of VINNA presented in this work are as follows:

(i) We provide the first publicly available open-source deep-learning pipeline for a combined subcortical segmentation as well as cortical, and white matter parcellation for newborn T1w or T2w MRIs.
(ii) We introduce a novel augmentation concept, which for the first time moves spatial augmentations into the network (instead of being performed outside). Our experimental results compare various spatial augmentation approaches side-by-side to isolate their effects.
(iii) We ensure fair comparisons throughout, for example, by fixed dataset splits, retraining methods under equal data and parameter settings, comparing architectures and setups with minimal differences, and quantifying real-world performance.
(iv) We, further, provide extensive comparison with state-of-the-art deep-learning methods (2D and 3D nnUNet) adapted for newborn segmentation (retrained on the same data) and present an extensive comparison to the publicly available newborn segmentation pipelines iBEAT and infantFS.

The specific application of VINNA to newborns (approximately 24–44 weeks post-menstrual age) will be made available as VINNA4neonates within our open source repository^1^ including Docker containers offering easy accessibility for the community.

### 1.1 Related work

While various reliable and sensitive traditional (Fischl et al., 2002; Friston et al., 2007; Jenkinson et al., 2012; Zhang et al., 2001) and fast deep-learning solutions exist (Billot et al., 2020; Chen et al., 2018; Coupé et al., 2020; Henschel et al., 2020, 2022; Huo et al., 2019; Iglesias et al., 2021; Ito et al., 2019; McClure et al., 2019; Mehta et al., 2017; Roy et al., 2019; Sun et al., 2019; Wachinger et al., 2018) for adult whole-brain segmentation, application of these methods to younger ages is hampered by the significant differences in size, MRI contrast profiles, and rapidly changing postnatal anatomy that is challenging to model with static templates.

### 1.2 Traditional tools for infant segmentation

Infant-specific traditional atlas-guided tools (Beare et al., 2016; Cherel et al., 2015; Makropoulos et al., 2018; Prieto et al., 2019; Shi et al., 2010; Zöllei et al., 2020) are predominantly optimized for a specific age range, resolution, and modality. Further, they differ significantly in the number of segmented classes and structure definitions.

The more recent Infant Brain Extraction and Analysis Toolbox (iBEAT) V2.0 (Wang et al., 2023) is a combination of age-specific CNNs for tissue segmentation, traditional surface generation, and parcellation, based on atlas registration, into 34 regions following the Desikan-Killiany protocol (Desikan et al., 2006). It supports a large age range (0–6 years), and allows segmentation of both, T1w and T2w MRI. While multiple input resolutions are supported, iBEAT internally reorients and resamples each image to a standard format (RAS orientation and 0.8 mm isotropic resolution). Hence, it does not support native resolution segmentation and image interpolation is required to map segmentations back to the original input space. The resampling step is automatically included in the pipeline such that in- and output resolutions are flexible. Furthermore, in its publicly available docker pipeline,^2^ segmentation is limited to white matter (WM), gray matter (GM), and cerebrospinal fluid (CSF).

infantFreeSurfer (infantFS) (Zöllei et al., 2020), on the other hand, mimics the FreeSurfer (Fischl, 2012) processing pipeline for adults and processes images from the first 2 years postnatally. It supports anatomical segmentation into 32 classes based on multi-atlas label fusion strategy, including registration to the infantFreeSurfer training data set (de Macedo Rodrigues et al., 2015). The entire pipeline is publicly available^3^ and allows processing of T1w images at a resolution of 1.0 mm, where the atlas training data are defined. For newborns, T1w images often suffer from poor tissue contrast due to the underlying myelination process, aggravating accurate registration from the atlases onto individual brains. This age group can therefore be a challenge for infantFS’s mono-modality approach.

The dHCP minimal-processing-pipeline (Makropoulos et al., 2018) is an optimized framework for cortical and sub-cortical volume segmentation, cortical surface extraction, and cortical surface inflation, which has been specifically designed for high-resolution T2w MRIs of newborns (Hughes et al., 2017). Here, an Expectation-Maximization algorithm, including an atlas-based spatial prior term, labels 87 classes based on a modified version of the ALBERTs atlas (Gousias et al., 2012; Makropoulos et al., 2014). The segmentations include subcortical structures, cortical and WM parcellations. Due to the cubic increase in voxel size for high-resolution images, processing times are in the order of hours to days for a single subject. This is a common limitation among traditional methods.

### 1.3 Deep-learning for infant segmentation

#### 1.3.1 Newborns

Overall, networks for cortical parcellations and subcortical structure segmentations in newborns are limited. The few existing CNNs support a single modality (T2w), fixed resolution, and segment a single (Rajchl et al., 2017) or eight (Moeskops et al., 2015) tissue classes. One recent exception is the deep-learning based neuroimaging pipeline by Shen et al. (2023), which is trained with the dHCP data. Here, the authors propose a 3D multi-task deep learning model with a U-Net like architecture to segment structural T1w and T2w images on both thin and thick sliced images. Unfortunately, the network follows a fixed-resolution scheme (0.8 mm), it does not support native segmentation across resolutions commonly encountered in newborn cohorts, and it is not readily available online.

#### 1.3.2 Isointense phase

The vast majority of deep-learning tools focus on processing of images at the isointense phase around 6 months after birth (Ding et al., 2022; Dolz et al., 2020; Kumar et al., 2018; Nie et al., 2016; Pasban et al., 2020; Qamar et al., 2020; Zeng & Zheng, 2018; Zeng et al., 2023; Zhang et al., 2015). Via the iSeg-challenge (Sun et al., 2021; Wang et al., 2019); data for training and validation are conveniently available, partly explaining this predominance. While many interesting architectural solutions have arisen, the main focus of the works is the effective combination of information from both T1w and T2w images to generate a broad segmentation into CSF, GM, and WM. This modality combination is specifically important in the isointense phase, which is characterized by changes in the myelination strongly effecting the appearance of the recorded MRIs (Gui et al., 2012; Wang et al., 2015; Weisenfeld & Warfield, 2009). The inversion of the WM-GM signal results in extremely low tissue contrast. The newborn cohort, on the other hand, demonstrates good contrast between GM and WM, specifically on the T2w images. While the age difference is small, the two cohorts as well as the associated challenges are distinct and networks trained on the one cannot easily be applied to the other. Subsequently, neither resolution-independence nor the stronger variation of head positions is specifically accounted for in network solutions for the isointense phase.

#### 1.3.3 Cross-age generalizability

To address generalizability across different age groups, recent research has suggested optimized training strategies for neonates, such as multi-task learning of tissue segmentation and geodesic distances (Bui et al., 2019) or the use of inductive biases in the form of pre-trained weights (i.e., fine-tuning to the target domain) (Wang et al., 2022). Both approaches improve segmentation accuracy, but they are still limited in their generalizability. They require retraining and hence a sufficient amount of labeled data; additionally, they rely on private datasets, limiting their reproducibility. Recently, a contrast agnostic segmentation via synthetic images, originally proposed for adult brain segmentation (Billot et al., 2020; Iglesias et al., 2021), has also been adopted for infant segmentation (Shang et al., 2022). Unfortunately, the output resolution is fixed for the network, and native resolution segmentations are not supported. Furthermore, while the model was able to generalize across a broader age range, the synthetic images still differ considerably from real data and the network, therefore they underperformed compared to age-specific models trained on existing MRIs. It should be noted that the authors did not aim to generate realistic images but rather a better segmentation tool.

### 1.4 Resolution-independence and position transforms in deep learning

A general resolution-ignorant framework addressing position transforms via external augmentations is nnUNet (Isensee et al., 2021). This network has successfully been applied for a variety of segmentation tasks due to its inherent ability to construct optimal parameter settings based on the input data itself. It provides different network set-ups (2D, 3D, and a cascaded 3D approach for large images) as well as a number of external image augmentations, including random rotation, scaling, mirroring, and gamma transformation. Interestingly, while the trained network also follows a fixed-resolution scheme, pre- and post-processing automatically resamples between original image and network resolution. While native resolution segmentation is not supported, in- and output resolutions are not fixed and the method is therefore a valid alternative to our VINNA. Both, the 2D and 3D nnUNet with external augmentation (exA) therefore serve as a state-of-the-art baseline for the newborn segmentation task.

A siamese network for semi-supervised training of a network to become equivariant to elastic transformation has been proposed in a single-resolution setting (Bortsova et al., 2019). A dedicated loss function assures that segmentations are consistent under a given class of transformation applied first to the image, and second to the output. The approach therefore relies on external augmentation and applies the transformation in the image space (before and after a UNet). The proposed VINNA, on the other hand, is fundamentally different. It shifts this step into the network itself, hence creating an internal augmentation. Overall, the approach by Bortsova et al. (2019) does therefore assure consistency across transformations in the labeling space, while VINNA targets spatial consistency of the feature maps.

In spatial transformers (Jaderberg et al., 2015), transformations attempt to harmonize or re-orient the image into a better position. To this end, an affine transformation is implicitly learned via a dedicated localisation network. Subsequent application of the calculated coordinate grid resamples the source feature maps via bi-linear interpolation to the new position. While our approach shares grid calculation and interpolation within the network with spatial transformers, our internal augmentation approach is inherently different. First, spatial transformers do not diversify or augment feature maps, but rather try to reach a harmonized position with respect to the data seen during training. External augmentations are still necessary to expose the network to a wide data variety and approximate equivariance. Second, instead of a localization network, we directly determine the sampling-grid based on a transformation matrix, which allows for an explicit integration of knowledge about the image, such as the resolution. As a result, computational complexity is reduced while achieving the desired position diversification and resolution-independence.

## 2 Material and Methods

### 2.1 Datasets

As the largest publicly available newborn dataset with intensity images, accompanying subcortical segmentations as well as cortical and WM parcellations at the time of our experiments, we randomly assign participants from the dHCP cohort with corresponding T1w and T2w MRIs to the training, testing, and validation sets, while ensuring equal distribution of age and gender. Additionally, the Melbourne Children’s Regional Infant Brain (M-CRIB) atlas cohort serves as an independent testing set for external validation of the final method. Written informed consent to participate in this study was provided by the participants’ legal guardian or next of kin in accordance with the Institutional Review Board. Complete ethic statements are available at the respective study webpages.

#### 2.1.1 dHCP

The developing Human Connectome Project (Makropoulos et al., 2018) includes T1w and T2w MRIs of newborns imaged without sedation on a 3 T Philips Achieva scanner. It provides 0.5 mm isotropic de-faced scans of individuals imaged postnatally between 24 to 45 weeks post-menstrual age. Imaging data for 578 participants with matching T2w and T1w are selected. The original images were acquired in sagittal and axial slice stacks with in-plane resolution of 0.8 mm × 0.8 mm and 1.6 mm slices overlapped by 0.8 mm. Motion correction and super-resolution reconstruction techniques (Cordero-Grande et al., 2018; Kuklisova-Murgasova et al., 2012) created isotropic volumes of resolution 0.5 mm. All T1w scans follow the same inversion recovery multi-slice fast spin-echo protocol with TR 4.795 s, TE 8.7 ms, TI 1.740 s, and SENSE factor 2.27 (axial) / 2.56 (sagittal). The parameters for the T2w scans are TR 12 s, TE 156 ms, and SENSE factor 2.11 (axial) / 2.66 (sagittal). The full dataset is available online.^4^ In the present study, 318 quality-checked images are used for network training and 90 for validation. A total of 170 images are used in the final test set.

**Fig. 2.**
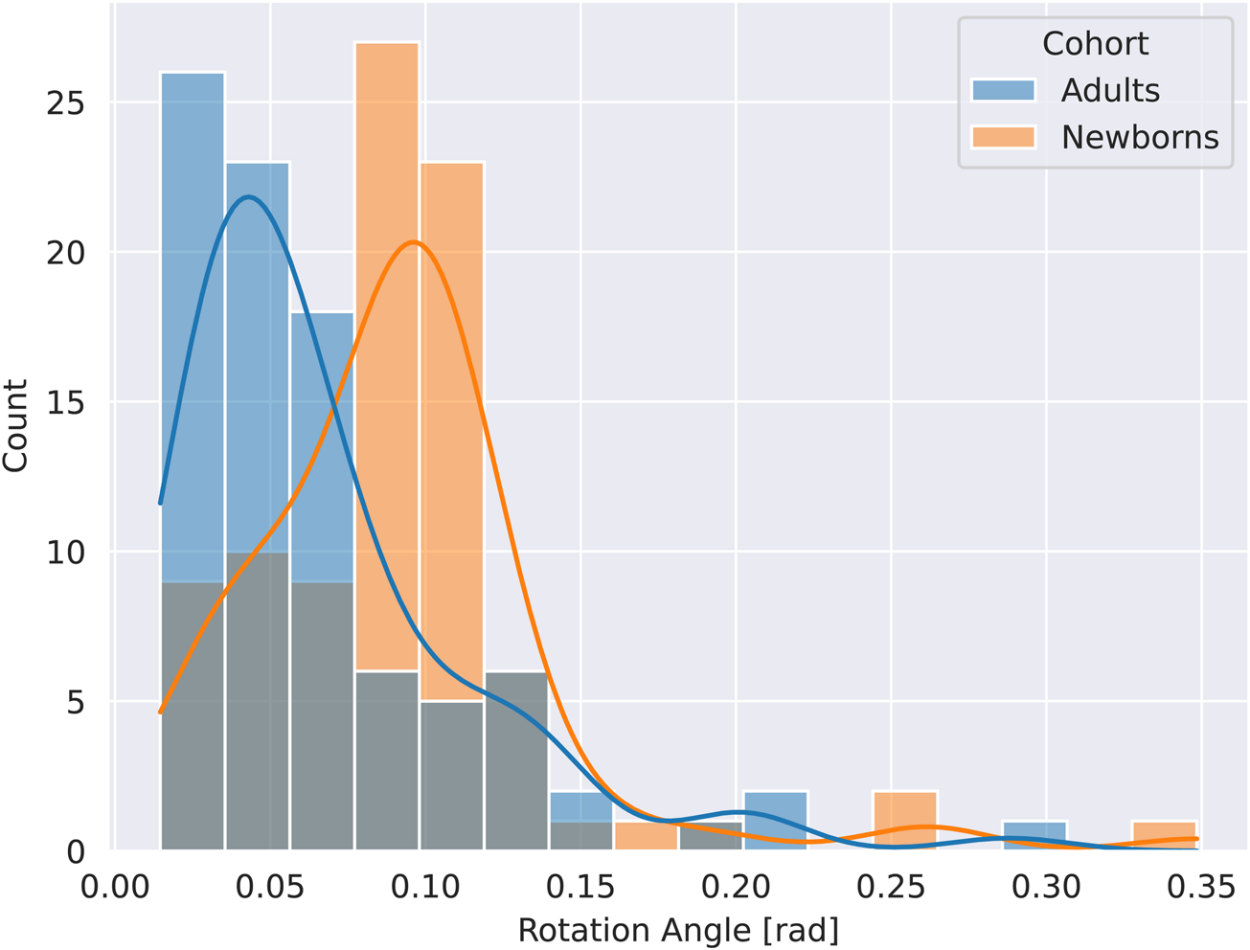
Comparison of variation in head position in newborns (orange) and adults (blue). Newborns show greater variation with respect to rotation angles. Rotation transformation is based on alignment of each individual subject to their midspace. N = 90 for both cohorts.

Even though the dHCP follows a well-defined protocol, standardization of positioning during scanning is still a challenge. As shown in Figure 2, inter-subject head position diversity in the newborn cohort is larger than an equally standardized adult cohort (HCP).

#### 2.1.2 M-CRIB

The Melbourne Children’s Regional Infant Brain (M-CRIB) atlas (Alexander et al., 2019) is constructed from 10 T2w MRI and corresponding manual segmentations of healthy term-born neonates (four females, six males) with post-menstrual age-at-scan between 40.29–43.00 weeks. The atlas comprises 94 neonatal brain regions compatible with the widely-used Desikan-Killiany-Tourville adult cortical atlas (Klein & Tourville, 2012). The T2w MRIs scanning protocols include the usage of a transverse T2 restore turbo spin echo sequence with 1.0 mm axial slices, a TR of 8.910 s, TE of 152 ms, flip angle of 120 degrees, Field Of View of 192 mm × 192 mm, and in-plane resolution of 1 mm (zero-filled interpolated to 0.5 mm × 0.5 mm × 1 mm). The T2w images are bias-corrected, skull-stripped, and resampled to 0.63 mm × 0.63 mm × 0.63 mm isotropic voxels. All 10 participants are used as an independent testing set for our external validation experiments.

### 2.2 Generation of reference segmentation with the dHCP-minimal-processing-pipeline

To imitate various resolutions and create the desired reference segmentations for training, we processed all raw dHCP MRIs with the dHCP-minimal-processing-pipeline (Makropoulos et al., 2018) at 1.0 mm, 0.8 mm, and 0.5 mm. The structure definitions follow the ALBERTs atlas (Gousias et al., 2012) with the subdivision of the WM and cortex proposed by Makropolus et al. (2014), resulting in a total of 87 structures (3 background labels, 20 subcortical regions, 32 cortical parcels, 32 WM parcels). We further lateralized the intracranial background based on the average Euclidean distance to neighboring labels, resulting in a final count of 88 labels. We provide a list of all segmentation labels used for training in the Appendix (see Appendix Table B). As the dHCP cohort includes both, T2w and a co-registered T1w MRIs, we trained dedicated networks for each modality. A manual quality check for all selected scans assured good overlap after the registration. Note that the dHCP-minimal-processing-pipeline relies on the original T2w images to create its segmentations, which are generally of higher quality in this collection.

### 2.3 Traditional infant segmentation tools

To evaluate VINNA against state-of-the-art traditional segmentation methods, we further process the testing set with the docker version of the iBEAT V2.0 pipeline (Wang et al., 2023) and infantFS (Zöllei et al., 2020).

#### 2.3.1 iBEAT

The iBEAT V2.0 pipeline (Wang et al., 2023) combines both traditional and deep-learning models to create tissue segmentations into three classes (GM, WM, and CSF), surface models, and cortical parcellations of the pial surface into 34 regions based on the Desikan-Killiany protocol (Desikan et al., 2006). For tissue segmentation, iBEAT uses seven age-specific CNNs trained on data for the representative age group (≤1, 3, 6, 9, 12, 18, and 24+ months of age). Neither the source code nor the training data and labels are publicly available. Hence, retraining of the models is not possible and comparisons are limited to the iBEAT pipeline output as is. For processing with iBEAT, submissions via a webserver^5^ or processing with a docker image^6^ are possible. The docker version does not currently support the cortical parcellations of the surface models. Due to the large number of participants, privacy concerns, and longer processing times when submitting via the webserver, we decided to use the docker version to process the T2w images of the testing set at the original 0.5 mm resolution. The resulting 3-label tissue segmentations form the basis for comparison to the other tools in this paper.

#### 2.3.2 infantFS

To allow comparison of segmentation performance to VINNA, all available T1w images from the dHCP testing set are processed with infantFS with default settings. The neuroimaging pipeline infantFS creates surface models, anatomical segmentations, and cortical parcellations based on the Desikan-Killiany-Tourville atlas (Klein & Tourville, 2012) for 0- to 2-year-old infants akin to the version for adults (FreeSurfer (Fischl, 2012)). The tool runs on T1w MRIs at a resolution of 1.0 mm (non-conforming images are resampled). infantFS relies on a registration-based multi-atlas label fusion strategy and returns an anatomical segmentation into 32 classes, including two labels for GM and WM.

#### 2.3.3 Label harmonization

**Fig. 3.**
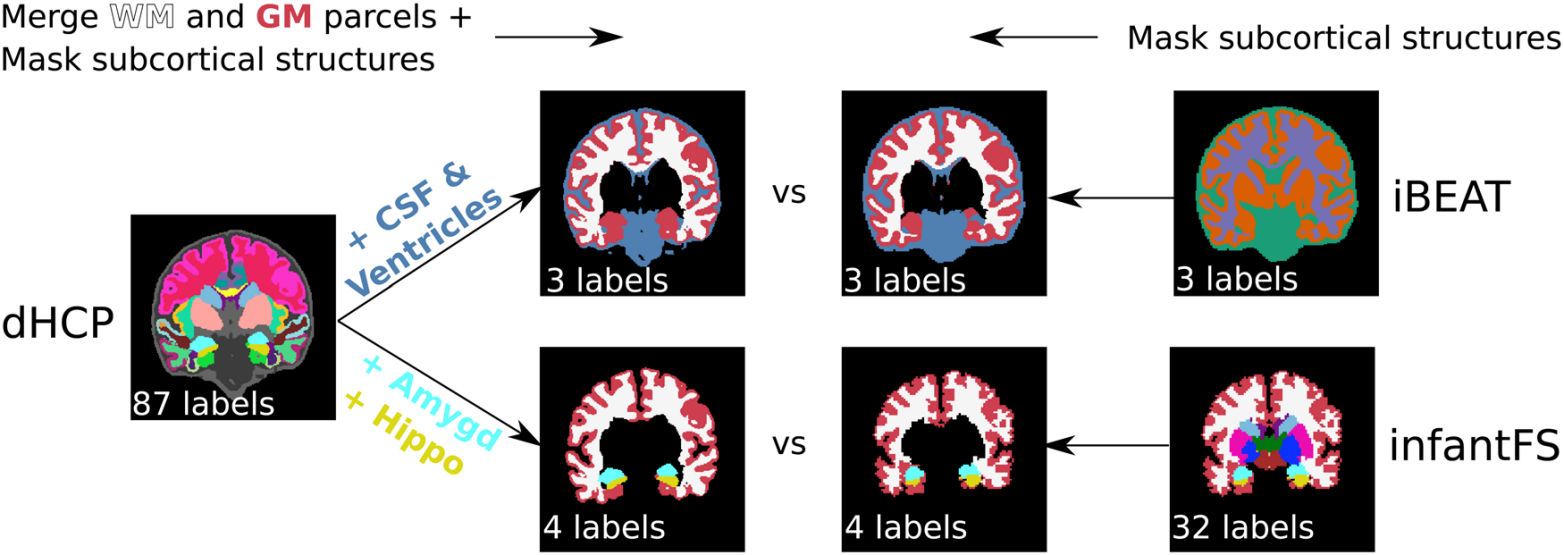
Harmonization of inconsistent label protocols between iBEAT, infantFS, and dHCP. Reduction of the original dHCP segmentation from 88 labels (left) by first merging all cortical parcels to cortex, WM parcels to WM and removal of subcortical structures, is followed by addition of CSF and ventricles (top) or amygdala and hippocampus (bottom) for comparison to iBEAT (3 labels: CSF (blue), WM (white), and GM (red), top second from left top) or infantFS (4 labels: WM (white), cortex (red), hippocampus (yellow), and amygdala (blue), bottom second from left), respectively. For iBEAT (right top), all GM and WM are modified using the dHCP segmentation for the subcortical structures, resulting in three labels for the final mapped version (3 labels, top second from right). For infantFS (right bottom), all structures except WM, cortex, hippocampus, and amygdala are removed (4 labels, bottom second from right).

As the dHCP-ALBERTs atlas differs from the resulting segmentations of both iBEAT and infantFS, we merge, remove, and mask classes to reach an approximate consensus across predictions. Figure 3 shows the merging protocol on a representative participant with the original and mapped ground truth dHCP labels (left side) together with iBEAT (top right side) and infantFS (bottom right side). First, the 32 cortical and WM parcels from the dHCP ground truth segmentation are reduced to two labels (cortex and WM) (top left in Fig. 3). For iBEAT, the WM additionally includes the corpus callosum while GM also encompasses the hippocampus and amygdala. The CSF label corresponds to the union of lateral-ventricles and CSF in the dHCP-ALBERTs atlas. In the dHCP ground truth, these labels are consequently merged to create the final three classes (GM, WM, and CSF; top second to left image). All other subcortical structures without a single possible assignment to GM, WM, or CSF are masked in the iBEAT prediction using the dHCP ground truth (Fig. 3, top right two images). For infantFS, the hippocampus and amygdala label remain, while individual cortex and WM parcels of the dHCP ground truth are merged (Fig. 3, bottom left images). Hence, in the infantFS predictions the following four labels remain: cortex, WM, hippocampus, and amygdala (Fig. 3, bottom two images to the right).

### 2.4 Network architectures

#### 2.4.1 Macro architecture

**Fig. 4.**
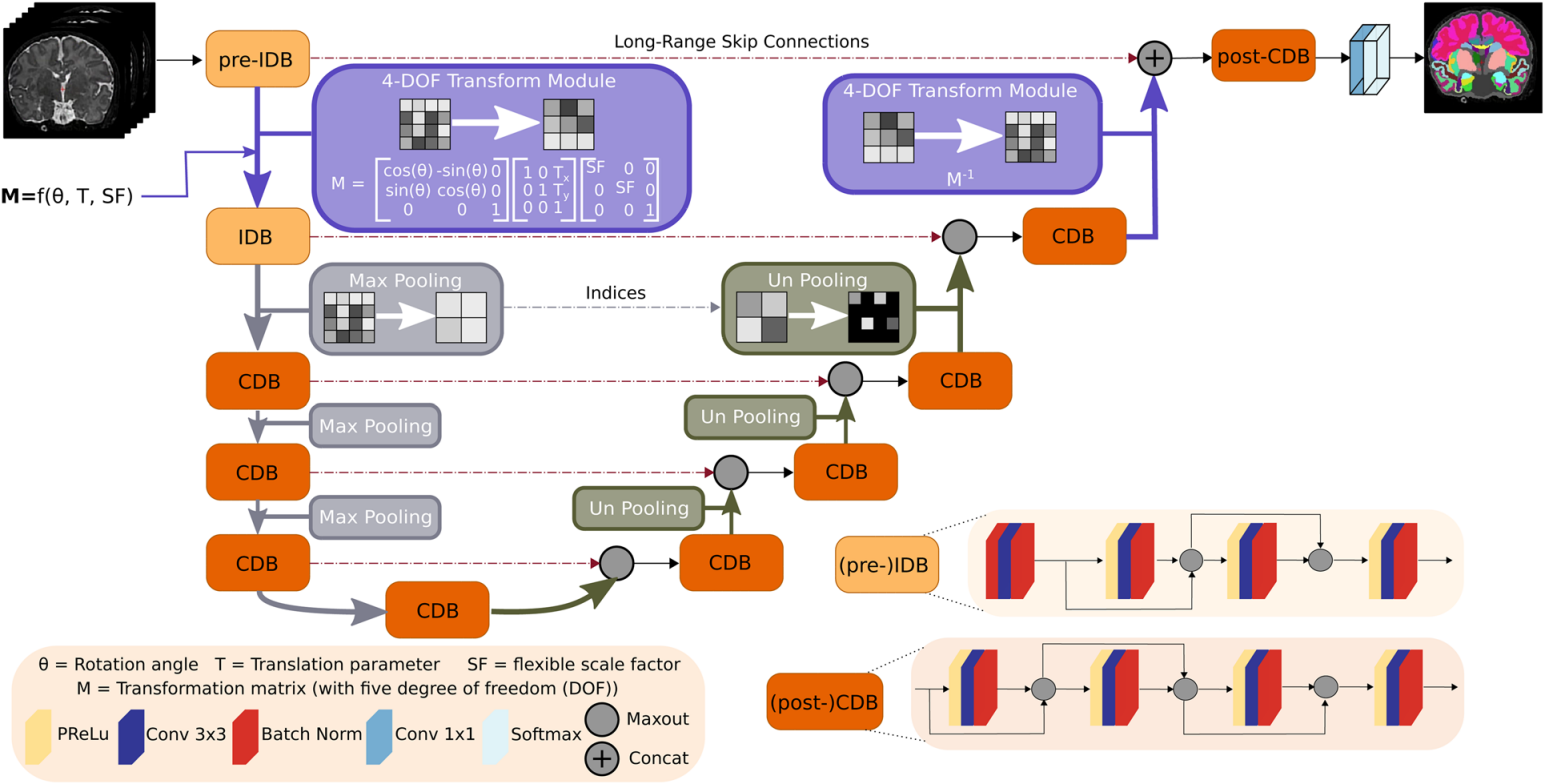
Network-integrated position variation and scaling normalization in VINNA. Flexible transitions between resolutions and head positions become possible by replacing (un)pooling with our network-integrated 4-DOF transform module (purple) after the first input dense block in the encoder (pre-IDB) and before the last competitive dense block in the decoder (post-CDB). A transformation matrix composed of rotation angle θ, translation parameter T, and the scaling factor SF defines the feature alterations. Scale transitions between the other competitive dense blocks (CDB) remain standard MaxPool and UnPool operations. Each CDB is composed of four sequences of parametric rectified linear unit (PReLU), convolution (Conv), and batch normalization (BN). In the first two encoder blocks ((pre)-IDB), the PReLU is replaced with a BN to normalize the inputs.

Figure 4 shows the macro architecture for VINNA. While the proposed 4-DOF transform module (purple) can, in theory, be included in any UNet-like architecture, we use the same basic setup for all trained models to assure maximum comparability (i.e., same number of parameters, same kernel sizes, etc.). The parameter-equal CNN is referred to as CNN*. CNN*, VINN, and VINNA, all contain an encoder and decoder consisting of five competitive dense blocks, respectively, which are separated by a bottleneck layer. In the encoder, maxpooling operations rescale the feature maps at each level by one half between the blocks using a pooling kernel of size 2×2 and stride 2. In contrast, index-unpooling doubles the feature map size in the decoder. Skip connections between the blocks at each level allow the gradient to flow efficiently. In CNN* (Henschel et al., 2022), pooling and unpooling operations transition between all levels (i.e., the purple block in Figure 4 is substituted with the gray maxpooling/unpooling operation). In VINN (Henschel et al., 2022), the first layer pooling and unpooling operation is replaced with a resolution-normalization module. This network-integrated flexible interpolation step allows transitions between resolutions without restrictions to pre-defined fixed voxel sizes, both during training and inference. Hence, images can be processed at their native resolution without prior resampling. Similar to spatial transformers (Jaderberg et al., 2015), the interpolation-based transition is divided into two parts: (i) calculation of the sampling coordinates (*grid generator*) and (ii) interpolation operation (*sampler*) to retrieve the spatially transferred output feature maps. Here, the sampling calculation relies only on the scale factor *SF*: the quotient of the resolution information of the inner normalized scale Res_inner_, a tune-able hyperparameter set to 0.8 mm throughout our experiments, and the input image Res_native_. The addition of parameter α sampled from a Gaussian distribution with parameters sigma = 0.1 and mean = 0 slightly augments the scale factor SF ($SF={\text{Res}}_{\text{inner}}/{\text{Res}}_{\text{native}}+\alpha$), introduces small resolution variations to the sampling, and increases the robustness of the latent space interpolation. Specifically, the presence of alpha allows for augmentations at the actual anatomical rather than the voxel size as the normalization of the resolution inside VINN disentangles perceived voxel versus actual structure size differences.

#### 2.4.2 Network-integrated 4-DOF transform module

In VINNA, the transition is implemented via the new network-integrated 4-DOF transform module shown in purple in Figure 4. Here, the sampling coordinate calculation is based on a transformation matrix $M\in {\text{R}}^{3\times 3}$ with four degrees of freedom, encoding not only scaling, but also in-plane rotation, and translation. Parameters for the rotation angle θ∈ℝ and translation $T\;\in {\mathbb{R}}^{2}$ are randomly sampled on the fly during training. The scale factor *SF* is calculated as in VINNs resolution-normalization module, that is, by dividing the inner normalized scale by the image resolution with augmentation by the parameter α. In the first transition step (Fig. 4, pre-IDB to IDB), the affine per-channel mapping M:U→V samples the input feature maps $U\in {\mathbb{R}}^{H_{\text{native}}\times W_{\text{native}}}$ to the output feature maps $V\in {\mathbb{R}}^{H_{\text{inner}}\times W_{\text{inner}}}$. In the final transition step (Fig. 4, competitive dense block (CDB) to post-CDB), this spatial transformation is reversed by using the inverse transformation matrix $M^{-1}:V\to U$. The interpolation itself is performed by applying a sampling kernel to the input map U to retrieve the value at a particular pixel in the output map V. The sampling is identical for each channel, hence conserving spatial consistency.

#### 2.4.3 Network blocks

##### 2.4.3.1 Competitive dense block (CDB) design

In VINNA, a CDB is formed by repetitions of the basic composite function consisting of a probabilistic rectified linear unit (pReLU) activation function, a 3×3 convolution, and a batch-normalization (BN). Feature competition within the block is achieved by using maxout (Goodfellow et al., 2013) instead of concatenations (Jégou et al., 2017) in the local skip connections. The maxout operation requires normalized inputs and is therefore always performed after the BN (see position of maxout in CDB design in Fig. 4).

##### 2.4.3.2 Input competitive dense block (IDB) design

In contrast to the described CDB, the first two network blocks follow a different order of operation. Here, the raw inputs are normalized by first passing them through a BN-Conv-BN combination before adhering to the original composite function scheme (Conv-BN-pReLU) (see Fig. 4, IDB).

##### 2.4.3.3 Pre-IDB

The first encoder block in VINNA called pre-IDB (see Fig. 4) transfers image intensity information from the native image to the latent space and encodes voxel size and subject-space-dependent information before the internal interpolation step. The composite function scheme is identical to the IDB, and the added prefix simply allows differentiation of the block placements.

##### 2.4.3.4 Post-CDB

Akin to the pre-IDB, an additional CDB block in the decoder merges the non-interpolated feature information returned from the pre-IDB skip connection and the upsampled feature maps from the network-integrated 4-DOF transform modules. A concatenation operation combines both feature maps, before passing them to a standard CDB block (see Fig. 4, (post-)CDB). After the final 1×1 convolution, a softmax operation returns the desired class probabilities.

### 2.5 Loss function

All networks are trained with a weighted composite loss function of logistic loss and Dice loss (Roy et al., 2017) combined with the high-resolution specific weighting from VINN (Henschel et al., 2022). In short, erosion and dilation of the cortex labels creates a binary mask of the outer cortex, small WM strands, and deep sulci. Wrong predictions in these areas result in a higher loss, hence guiding the network to focus on areas particularly affected by partial volume effect (PVE). The original publications’ ablation experiments evaluated the impact of the different function elements: the logistic loss and Dice loss combination improves overall segmentation performance (Roy et al., 2017), while the high-resolution weighting leads to higher segmentation accuracy on the cortical parcels (Henschel et al., 2022).

If we consider $p_{l,i}\left(x\right)$ as the estimated probability of pixel i that belongs to class l,​y, as the corresponding ground truth probability, and $\omega_{i}$ as the associated weight given to the pixel i based the loss function can be formulated as

$$ℒ=\underset{\text{Logistic loss}}{\underset{︸}{-\sum_{l,i}\omega_{i}y_{l,i}\text{log}\;p_{l,i}\left(x\right)}}-\underset{\text{Soft Dice loss}}{\underset{︸}{\sum_{l}\frac{2\sum_{i}\;p_{l,i}\left(x\right)y_{l,i}}{\sum_{i}\;p_{l,i}\left(x\right)+\sum_{i}y_{l,i}}}}$$ (1)

with $\omega_{i}=\omega_{\text{median freq.}}+\omega_{\text{gradient}}+\omega_{\text{GM}}+\omega_{\text{WM/Sulci}}$.

Here, $\omega_{\text{median freq}}$. represents median frequency balancing addressing the class imbalance and $\omega_{\text{gradient}}$ boundary refinement through a 2D gradient vector (Roy et al., 2017), while $\omega_{\text{GM}}$ and $\omega_{\text{WM/Sulci}}$ assign higher weights to PVE-affected areas (Henschel et al., 2022).

### 2.6 View aggregation

In order to account for the inherent 3D geometry of the brain, we adopt the 2.5D view aggregation scheme from (Henschel et al., 2020, 2022) for CNN*, VINN, and VINNA. In short, we train one network instance per anatomical plane and calculate a weighted average of the resulting softmax probability maps. The weight of the sagittal predictions is reduced by one half compared to the other two views to account for the missing lateralization in the sagittal view. In this plane, the network predicts 23 instead of 88 classes.

### 2.7 Augmentations

#### 2.7.1 External augmentation (exA)

The current state-of-the-art approach to introduce robustness to position changes into neural networks is extensive external augmentation (see Fig. 1B). Therefore, we contrast our proposed network-integrated 4-DOF transform module against this approach. We use random transforms with rotation parameters sampled from a uniform distribution of the predefined range -180° to 180° and translation by 0 to 15 px to augment images during the training phase and interpolate linearly. For CNN* and nnUNet, augmentation also includes sampling of scaling parameters from a uniform distribution of the predefined range 0.8 to 1.15. VINN’s resolution-normalization module makes this step obsolete. Every minibatch hence consists of a potentially transformed MRI (using bi-linear interpolation) and a corresponding label map (using NN sampling). By exposing a network to a large variety of possible image positions during training, orientation-robustness can be achieved. All external augmentation routines are implemented using torchIO (Pérez-García et al., 2021).

#### 2.7.2 Image intensity augmentation

To allow generalization outside of the dHCP cohort, we apply a number of intensity or texture augmentations on the fly to the training batch, namely bias field changes, random gamma alterations, ghosting, spiking, blurring, and Gaussian noise. Each batch sampled from the original data is transformed by any of the operations above with a probability of 0.4. As before, all augmentations are implemented using torchIO.

### 2.8 Evaluation metrics

We use the Dice Similarity Coefficient (DSC) (Dice, 1945; Sørensen, 1948) and Average Surface Distance (ASD) to compare different network architectures and modifications against each other, and to estimate similarity of the predictions with a number of previously unseen scans. Both are standard metrics to evaluate segmentation performance. We establish improvements by statistical testing (Wilcoxon signed-rank test (Wilcoxon, 1945) after Benjamini-Hochberg correction (Benjamini & Hochberg, 1995) for multiple testing) referred to as “corrected p” throughout the paper.

### 2.9 Training setup for all models

#### 2.9.1 Training dataset

For training, we select 318 representative participants from the dHCP cohort. Resolutions are equally represented with 106 MRIs at 1.0 mm, 0.8 mm, and 0.5 mm, respectively. Empty slices are filtered from the volumes, leaving on average 137 single view planes per subject and a total training size of at least 20k images per network. The original nnUNet does not filter the volumes. The 3D version uses 3D patches instead of 2D slices. The parameters are automatically determined by nnUNet to guarantee an ideal set-up for the given segmentation task. Otherwise, we train all directly compared networks (CNN*, VINN, VINNA, 2D nnUNet, and 3D nnUNet) under the same conditions.

#### 2.9.2 Training parameters

We implement and train independent models to convergence (early stopping) for the coronal, axial, and sagittal planes with PyTorch (Paszke et al., 2017), using one NVIDIA V100 GPU with 32GB RAM. During training, the modified Adam optimizer (Loshchilov & Hutter, 2019) is used with a learning rate of 0.001. Using a cosine annealing schedule (Loshchilov & Hutter, 2017) with warm restarts, the learning rate is adapted after initially 10 epochs. The epoch offset is subsequently increased by a factor of two. The momentum parameter is fixed at 0.95 to compensate for the relatively small mini batch size of 16 images for CNN*, VINN, and VINNA. For nnUNet, the optimal batch-size is automatically determined (Isensee et al., 2021). For the given segmentation problem, the 2D nnUNet uses a batch size of 128 while the 3D version relies on a smaller batch size of 128. To ensure a fair comparison, all networks (CNN*, VINN, VINNA, 2D nnUNet, and 3D nnUNet) have been trained under equal hardware and hyper-parameter settings otherwise. A comparing table is available in the Appendix (Section A.4).

## 3 Results

We group the presentation of results into three blocks: 1. ablative architecture improvements to determine the best performing module for orientation and position transformation (Section 3.1), 2. performance analysis to comprehensively characterize the advantages of VINNA with respect to state-of-the-art traditional atlas- and deep-learning-based methods (Section 3.2), and 3. external validation on M-CRIB (Alexander et al., 2019) to asses generalizability and performance with respect to manual labels (Section 3.3). Following best practice in data-science, we utilize completely separate datasets during the evaluations: the validation set for Section 3.1 (Appendix Table A: Validation), and various test sets for Sections 3.2 and 3.3 (Appendix Table A: Testing). This avoids data leakage and ensures that training, method design decisions, and final testing do not influence each other, which could otherwise lead to overly optimistic results (overfitting).

### 3.1 External augmentation versus network-integrated 4-DOF transform module in VINNA

As high variances with respect to head orientations and spatial resolutions are common in newborns and are likely to be underrepresented in the limited available data cohorts, we first compare multiple approaches for extension of the training distribution for accurate (sub)millimeter newborn whole-brain segmentation. Traditionally, external data augmentation (exA), such as scaling, rotation, or translation, addresses this problem by interpolating both, the image and label maps, to a new, random position during training. Due to the discrete nature of the label maps, lossy NN interpolation cannot be avoided, unless the transformations are applied to the one-hot-encoded logits (soft-loss). We therefore evaluate both, the traditional exA and soft-loss implementation (referred to as exA (soft)). Our new 4-DOF transform module in VINNA internally emulates possible head transformations and acts directly on the encoded feature maps. To evaluate effectiveness of the exA versus VINNA, we compare VINNA with parameter-identical CNN*, VINN, and VINNA equipped with exA. Each subsequent improvement in segmentation performance is confirmed by statistical testing (corrected p < 0.05).

**Fig. 5.**
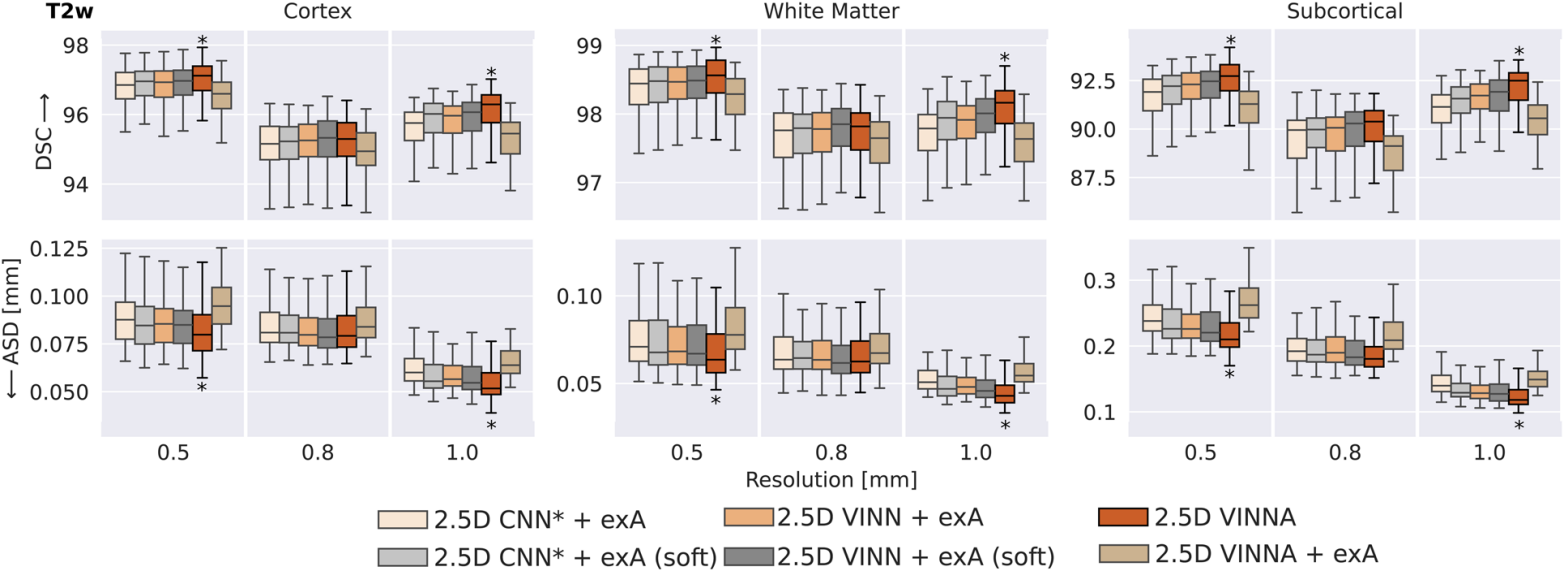
Comparison of approaches for external and internal spatial augmentation on T2w images: Our VINNA method—with the 4-DOF transform module—(fifth box in the group) outperforms both state-of-the-art approaches with external Augmentation (CNN* + exA and VINN + exA, first and third box) in Dice Similarity Coefficient (DSC, upper row) and average surface distance (ASD, lower row). This performance advantage is significant (corrected $p\;<\;10^{-9}$) and consistent across resolutions and structures. Combining VINNA with external augmentation (VINNA + exA, last box) reduces performance. When avoiding nearest-neighbor label interpolation by applying exA on one-hot encoded logits (+exA (soft)) instead of the label maps, performance improves for both CNN* and VINN (second box and fourth box, respectively). VINNA with the internal 4-DOF module outperforms the soft-loss methods on 1.0 and 0.5 mm by a significant margin (corrected $p\;<\;10^{-9}$, indicated with*). exA: external Augmentation.

In Figure 5, we compare the model performance of six approaches: CNN* with exA and exA (soft), (Section 2.4, CNN* + exA, left box; CNN* + exA (soft), second box from the left), VINN with exA and exA (soft) (Section 2.4, VINN + exA, third box from the left; VINN + exA (soft), fourth box from the left), VINNA with the new 4-DOF transform module (VINNA, second box from the right), and finally VINNA with exA (VINNA + exA, right box). The analysis of the DSC (top) and ASD (bottom) is grouped for three groups of structures (cortex averages 32 labels, WM averages 32 labels, and subcortical structures average 20 labels) and three resolutions (from left to right 0.5 mm, 0.8 mm, and 1.0 mm). We present performance for T2w MRIs, but we found the same ranking for T1w MRIs.^7^

Looking at the T2w segmentation and focusing on the different resolutions, the differences between the approaches are largest on the subcortical structures. The CNN* with exA reaches an average DSC of 91.91, 89.95, and 91.14 and an ASD of 0.238 mm, 0.192 mm, and 0.193 mm for input data of 0.5 mm, 0.8 mm, and 1.0 mm, respectively. The slight reduction in performance for 0.8 mm resolution consistently occurs for all evaluated models and is probably caused by the necessary image resampling from the original resolution of 0.5 mm and subsequent reprocessing with the dHCP-pipeline (Section 2.2). Interpolation from 0.5 mm to 1.0 mm results in a well-aligned grid due to the even division by factor 2 (8 voxels get averaged into a single larger voxel). Resampling to 0.8 mm, on the other hand, requires an uneven interpolation grid with weighted averages and original voxels that contribute to multiple larger voxels. This more challenging setting could result in the slightly reduced segmentation performance. Optimization of the architecture design towards multi-resolution (VINN, Section 2.4) leads to significant improvement in the DSC and ASD across the cortical, WM, and subcortical structures (Fig. 5, VINN + exA). Particularly, the subcortical segmentations are improved by around 0.5%. Importantly, the internal 4-DOF transform module (VINNA), which avoids label interpolation all together, further reduces the error by one half and increases segmentation performance significantly compared to both CNN* and VINN with exA. This effect is consistent across all structures and resolutions. Specifically, the ASD at the high-resolution benefits from the new module. Here, performance can be improved by 4.47% on the cortex, 5.19% on the WM, and 5.89% on the subcortical structures. For the lower resolution, the improvement on the cortex and WM is slightly lower (around 2%) while the subcortical structures benefit from the 4-DOF transform module similarly to the 0.5 mm resolution experiments.

The soft-loss, implementing augmentations for the label maps via linear interpolation of the one-hot encoded feature maps, does improve performance compared to the nearest-neighbor based external augmentation for both CNN* and VINN (see CNN*/VINN + exA versus CNN*/VINN + exA (soft)). The subcortical structures improve the most (0.27% DSC and 5.1% ASD for CNN* + exA versus + exA (soft); 0.21% and 2.35% ASD for VINN + exA versus + exA (soft)), followed by the cortex (0.15% DSC and 3.7% ASD; 0.07% DSC and 1.8% ASD). The improved performance without nearest-neighbor interpolation further strengthens our recommendation to avoid this type of label interpolation wherever possible. The proposed VINNA architecture outperforms VINN + exA (soft) on the 0.5 and 1.0 mm resolution by 0.17% and 0.35% DSC and 5.27% and 6.23% ASD, respectively. The subcortical structures benefit the most with an average improvement by 0.34% DSC and 4.39% ASD, respectively. On the 0.8 mm resolution, no significant difference is detectable between the two approaches.

Overall, VINNA reaches the highest DSC and lowest ASD for the cortical structures (96.24, 0.079 mm), WM (98.18, 0.063 mm), and subcortical structures (91.87, 0.190 mm) across all resolutions. The addition of exA to the framework (VINNA + exA, right box in each plot) again reduces performance. The DSC drops by 0.4%, 0.28%, and 1.15% on the cortex, WM and subcortical structures on average, while the ASD worsens by 4.75%, 3.92%, and 4.84%. Overall, results with VINNA are significantly better on 0.5 mm and 1.0 mm compared to all ablations on the validation set (corrected $p\;<\;10^{-6}$).

### 3.2 Comparison to state-of-the-art neonate segmentation tools

To evaluate how VINNA compares to state-of-the-art neonate MRI segmentation tools, namely nnUNet (3D and 2D), iBEAT, and infantFS, we take a closer look at the DSC and ASD on the testing sets.

#### 3.2.1 Comparison of deep-learning networks

**Fig. 6.**
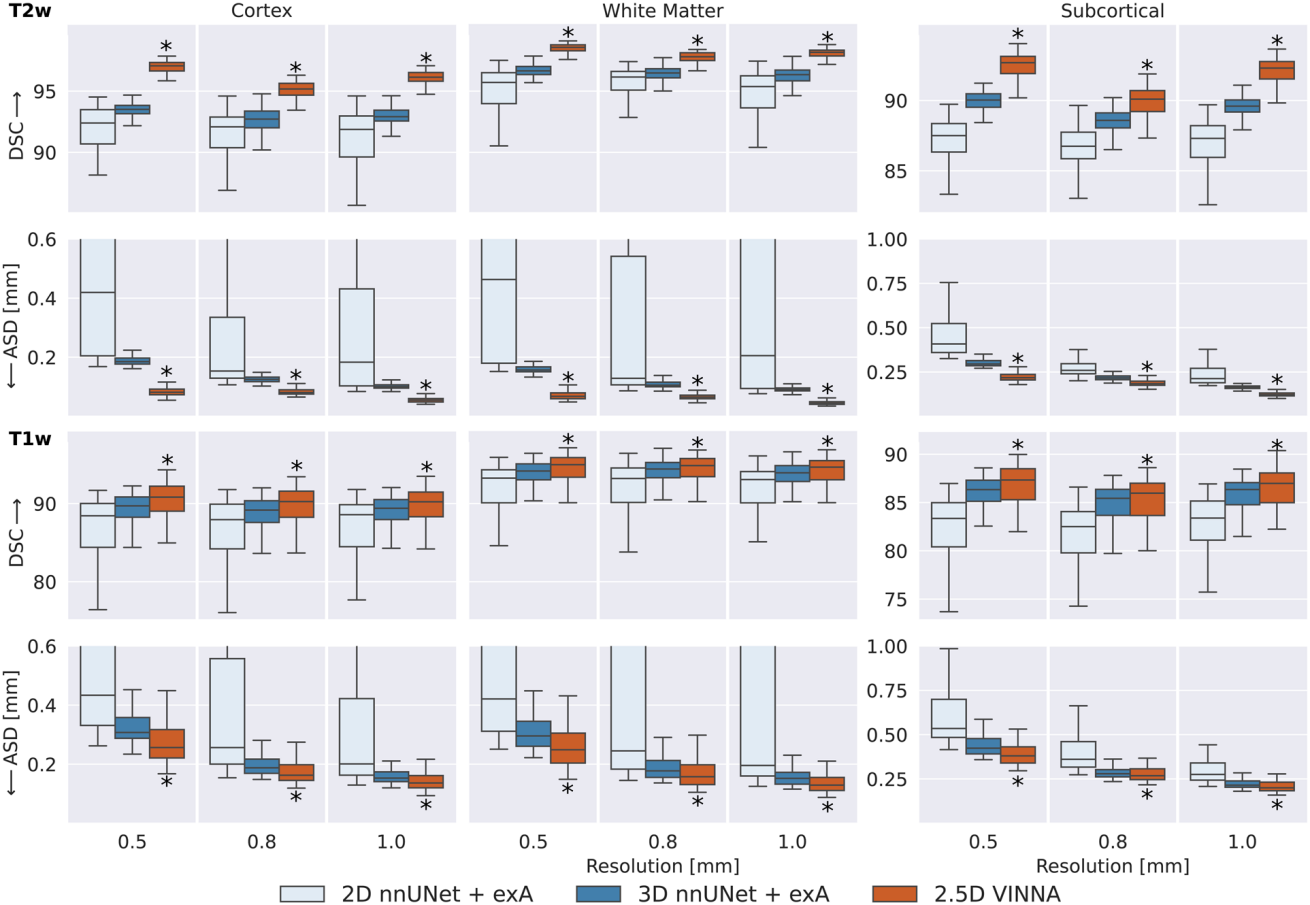
SOTA Segmentation performance: Our VINNA with the 4-DOF transform module (last box in group) outperforms the three state-of-the-art deep-learning approaches, 2D nnUNet + exA and 3D (first and second box) in Dice Similarity Coefficient (DSC, upper row) and average surface distance (ASD, lower row). This performance advantage is significant (corrected $p\;<\;10^{-10}$, indicated with *) and consistent across three resolutions (0.5 mm, 0.8 mm, and 1.0 mm), two modalities (T2w, top and T1w, bottom), and three structure groups (cortex, WM, and subcortical structures).

Figure 6 shows a detailed comparison of three different deep-learning based methods for neonate segmentation across modalities (T2w top, T1w bottom) and resolutions. All models are trained under equal parameter and dataset settings. Comparing performance between the two modalities shows that all models perform better on the T2w (top) than the T1w MRIs (bottom) across all structures. With VINNA (right box in each plot), the reduction is similar across all resolutions, with an average difference in DSC of 5.85, 3.47, and 5.16 on the cortex, WM, and subcortical structures. The ASD is, on average, improved by 0.09 mm when predicting on the T2w instead of T1w inputs. The nnUNet framework in 2D (left box) and 3D (second from left) has less improvement on the T2w images with an average difference between T1w and T2w of 3.75, 2.44, and 3.70 in DSC and 0.04 mm ASD on the aforementioned structures.

When comparing the four models, the 2D nnUNet + exA (left box) version performs worse than the 3D (second from left box), and 2.5D VINNA (right box) across all resolutions, structures, and modalities. Particularly notable are the large variations of 2D nnUNet + exA in prediction performance (large standard deviation) and large ASD (see Fig. 6), especially at the highest resolution (0.42 mm, for the cortex, 0.46 mm for WM and 0.41 mm for subcortical structures). This difference is less prominent in the DSC scores, but 2D nnUNet + exA also performs worst across all resolutions with respect to this metric (i.e., 92.39, 95.70, and 87.50 for a resolution of 0.5 mm). The 3D nnUNet + exA (second from left) improves accuracy by 24.5%, 12.3%, and 22.9% for ASD and 1.75%, 1.06%, and 1.64% for DSC across the three different resolutions. VINNA with its 4-DOF transform module (VINNA, right box) is again the best performing model, significantly outperforming all other networks. Compared to the 3D nnUNet + exA, ASD and DSC scores are significantly improved with the highest gain on the cortical structures (56%, 35%, and 45% ASD and 3.8%, 2.7%, and 3.5% DSC for 0.5 mm, 0.8 mm, and 1.0 mm, respectively).

**Fig. 7.**
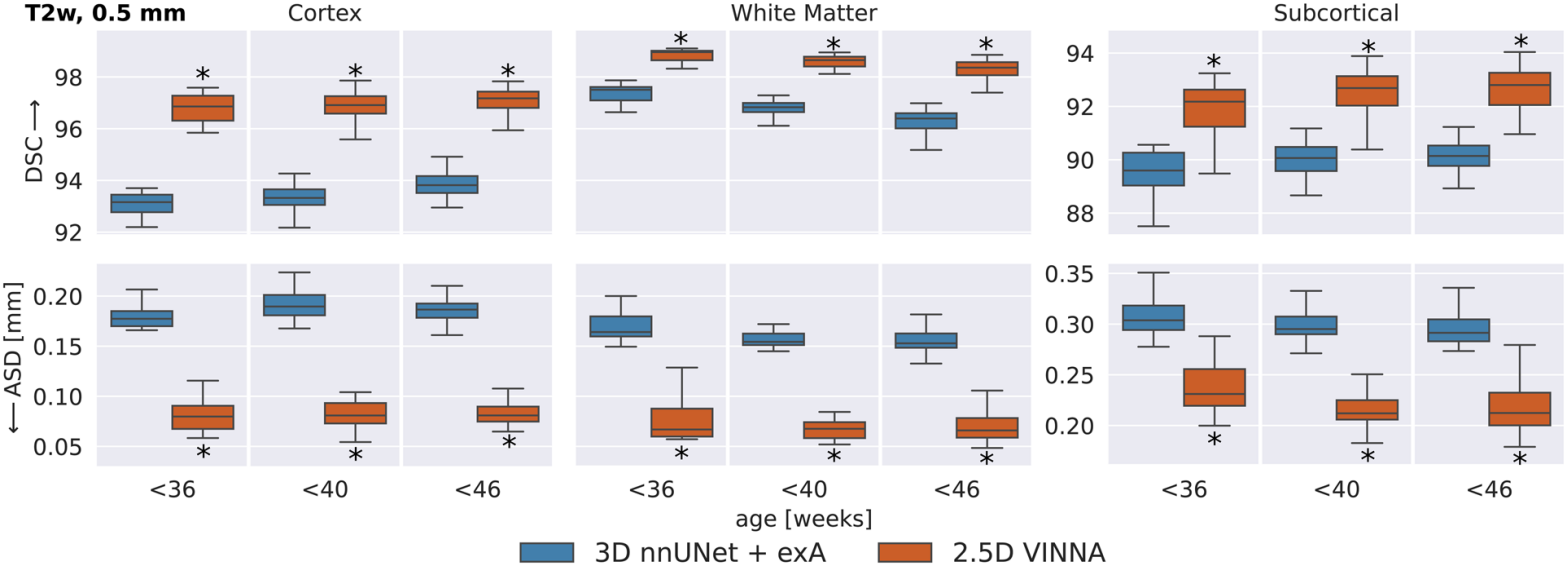
SOTA Segmentation performance across age groups: VINNA equipped with the 4-DOF transform module (right box) consistently outperforms state-of-the-art deep-learning approaches 3D nnUNet with external augmentation (+exA, left box) across four age groups. Improvement in dice similarity coefficient (DSC, top) and average surface distance (ASD, bottom) on T2w at 0.5 mm is significant (corrected $p\;<\;10^{-4}$, indicated with *) for <36, <40, and <46 week-old newborns.

To evaluate if this trend is consistent across age groups, the 0.5 mm MRI images are split into three approximately equal-sized groups based on the participants’ age-at-scan information (32–36, 36–40, and 40–46 weeks). Figure 6 shows DSC (top) and ASD (bottom) calculated for T2w (Fig. 7) for the 3D nnUNet + exA (left box) and VINNA with its 4-DOF transform module (right box) in each of the categories.

Consistent with the previous section, 3D nnUNet + exA reaches the weakest ASD and DSC across all age groups. On average, the VINNA with its 4-DOF transform module (right box) improves performance compared to nnUNet by 2.8%, 2.9%, and 2.8% DSC and 41.5%, 43.6%, and 43.1% ASD from the youngest (32–36) to the oldest (<46) age group and reaches a DSC of 96.86, 96.913, and 97.17 for the cortical structures, 98.96, 98.652, and 98.362 for the WM structures, and 92.18, 92.69, and 92.80 for the subcortical structures across all age groups (youngest to oldest) on the T2w MRIs (Fig. 7, top). Here, VINNA also reaches the lowest ASD (0.080 mm, 0.081 mm, and 0.081 mm for the cortical structures, 0.067 mm, 0.068 mm, and 0.066 mm for the WM structures, and 0.231 mm, 0.212 mm, and 0.212 mm for the subcortical structures). The results are significantly better compared to 3D nnUNet + exA (corrected $p\;<\;10^{-9}$). As seen by the increasing DSC and decreasing ASD, the younger age groups (<32−36) have proved to be more challenging to segment. For VINNA, the performance decreases most significantly for the subcortical structures (DSC by 1.82% and ASD by 20.09%) and least on the cortex (0.35% DSC and 4.65% ASD). This trend is consistent for the other two models. Assessment of qualitative differences between the 3D nnUNet + exA and VINNA on a representative participant at 40 weeks of age (Fig. 10) shows slight over-segmentation of the cortex and loss of small WM strands with the 3D nnUNet + exA (third row, second column, arrows). Overall, the segmentation with VINNA (fourth row) appears less smoothed and closer to the ground truth (second row).

#### 3.2.2 Comparison to iBEAT

**Fig. 8.**
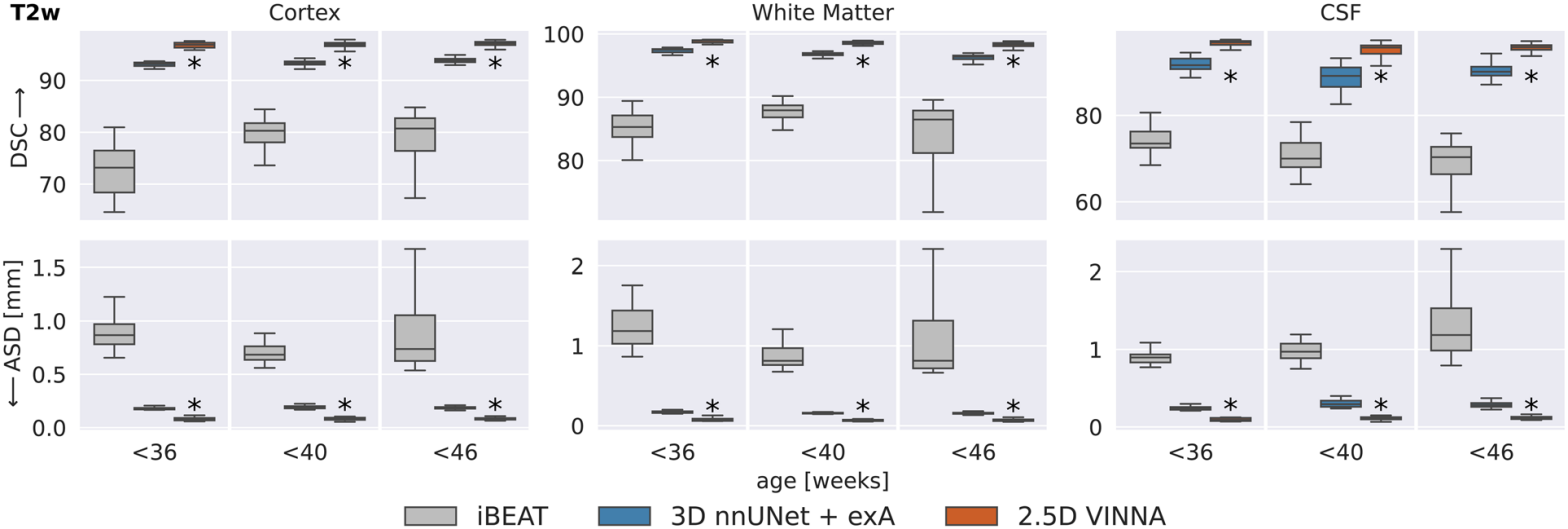
Deep-learning networks versus iBEAT: Similarity to the dHCP reference is higher with VINNA (third box) and 3D nnUNet + exA (second box) than iBEAT (first box) with respect to dice similarity coefficient (DSC, top) and average surface distance (ASD, bottom) on T2w MRIs at 0.5 mm across three age groups. Segmentation results with VINNA are significantly closer to dHCP (corrected $p\;<\;10^{-6}$, indicated with *) for CSF, GM, and WM. Note that iBEAT’s structure definition is not identical to the dHCP-ALBERTs atlas and analysis is based on harmonized, merged labels.

In Figure 8, the deep-learning models are compared to the docker version of iBEAT on the T2w images at 0.5 mm. iBEAT is officially designed for ages 0–6 years and the docker version we used for processing returns three labels (WM, GM, CSF). The definition of these labels is different from that of the dHCP-atlas (see Section 2.3.3), so we map the ground truth as well as the predictions from the deep-learning networks (nnUNet3D and VINNA) to be able to compare segmentation similarity. As described in Section 2.3, retraining the CNN part of iBEAT under same data and label definitions is not possible, as neither the source code nor the original training data is available online. Note, even though we do not need to interpolate, cross-protocol comparisons include atlas differences and may introduce additional errors due to the mapping. While results should be interpreted with the caveat that iBEAT uses a different atlas and training dataset than 3D nnUNet + exA and VINNA, the label harmonization allows an as-fair-as-possible comparison with this state-of-the-art method.

With respect to the mapped dHCP-reference segmentation, DSC (top) and ASD (bottom) are lower for iBEAT (left box in each plot) compared to the the deep-learning methods (3D nnUNet + exA, middle box, and VINNA, right box in each plot). Performance of iBEAT on the GM, WM, and CSF improves with age. GM and WM are closest to the reference at 36–40 weeks (DSC 80.29/87.94 and ASD 0.6840.813 mm), while CSF peaks at 32–26 weeks (73.49 and 0.896 mm). The 3D nnUNet + exA and VINNA show a similar trend, but performance is more consistent. As mentioned before, the differences are not necessarily due to wrong predictions made by iBEAT. Looking at the qualitative comparison in Figure 10, differences appear small, with iBEAT (third row) missing a few WM strands (arrow) and slightly over-segmenting the cortex compared to the mapped ground truth (second row).

#### 3.2.3 Comparison to infantFS

**Fig. 9.**
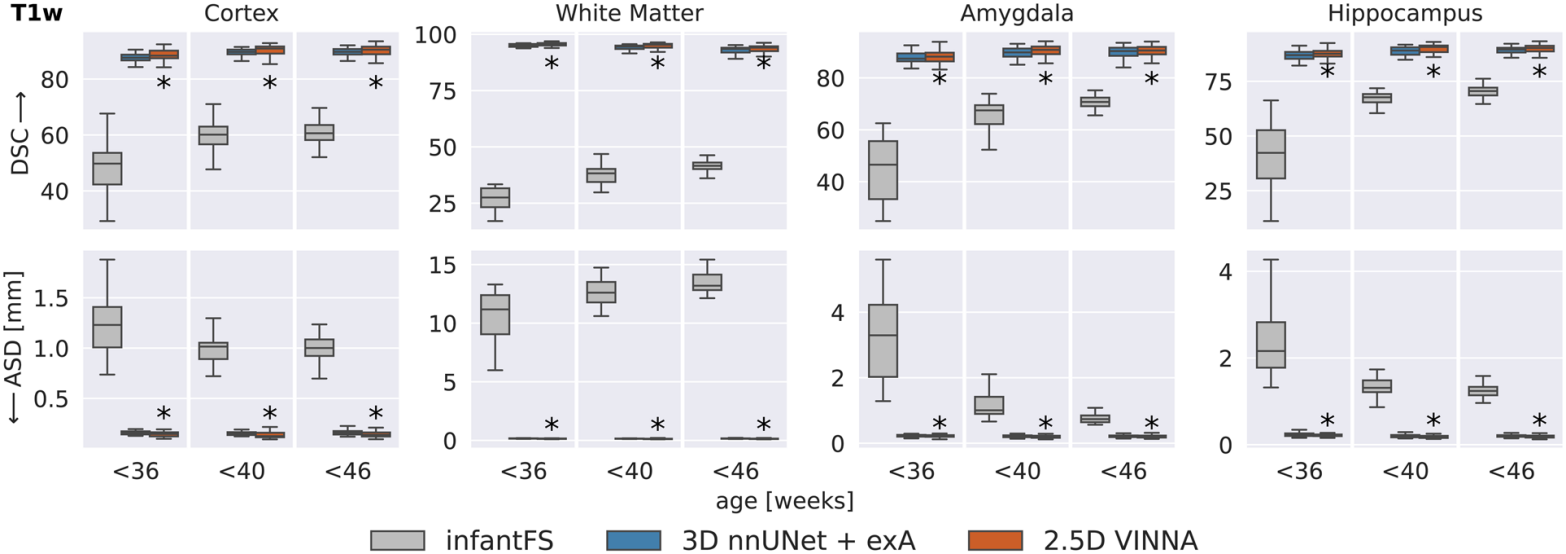
Deep-learning networks versus infantFS: VINNA with the 4-DOF transform module (third box) and 3D nnUNet + exA (second box) are closer to the dHCP reference than infantFS (first box) on T1w at 1.0 mm, the supported modality and resolution for infantFS. Dice Similarity Coefficient (DSC, top) and average surface distance (ASD, bottom) significantly improve with VINNA (corrected $p\;<\;10^{-6}$, indicated with *) on the cortex, WM, hippocampus, and amygdala across all age groups. Note that definition of subcortical structures differs in infantFS and predictions are harmonized to allow comparison to the deep-learning models.

Figure 9 shows performance comparison between infantFS (left box) and the deep-learning methods, 3D nnUNet + exA (middle box) and VINNA (left box), but in contrast to previous evaluations on T1w images at 1.0 mm, the operating resolution and modality of infantFS. Note that the infantFS labels are also different from those of dHCP and the ground truth labels must be mapped (see Section 2.3.3 for details). As for iBEAT, the cross-protocol comparison can put the traditional method at an unfair disadvantage and results should be considered with this caveat.

**Fig. 10.**
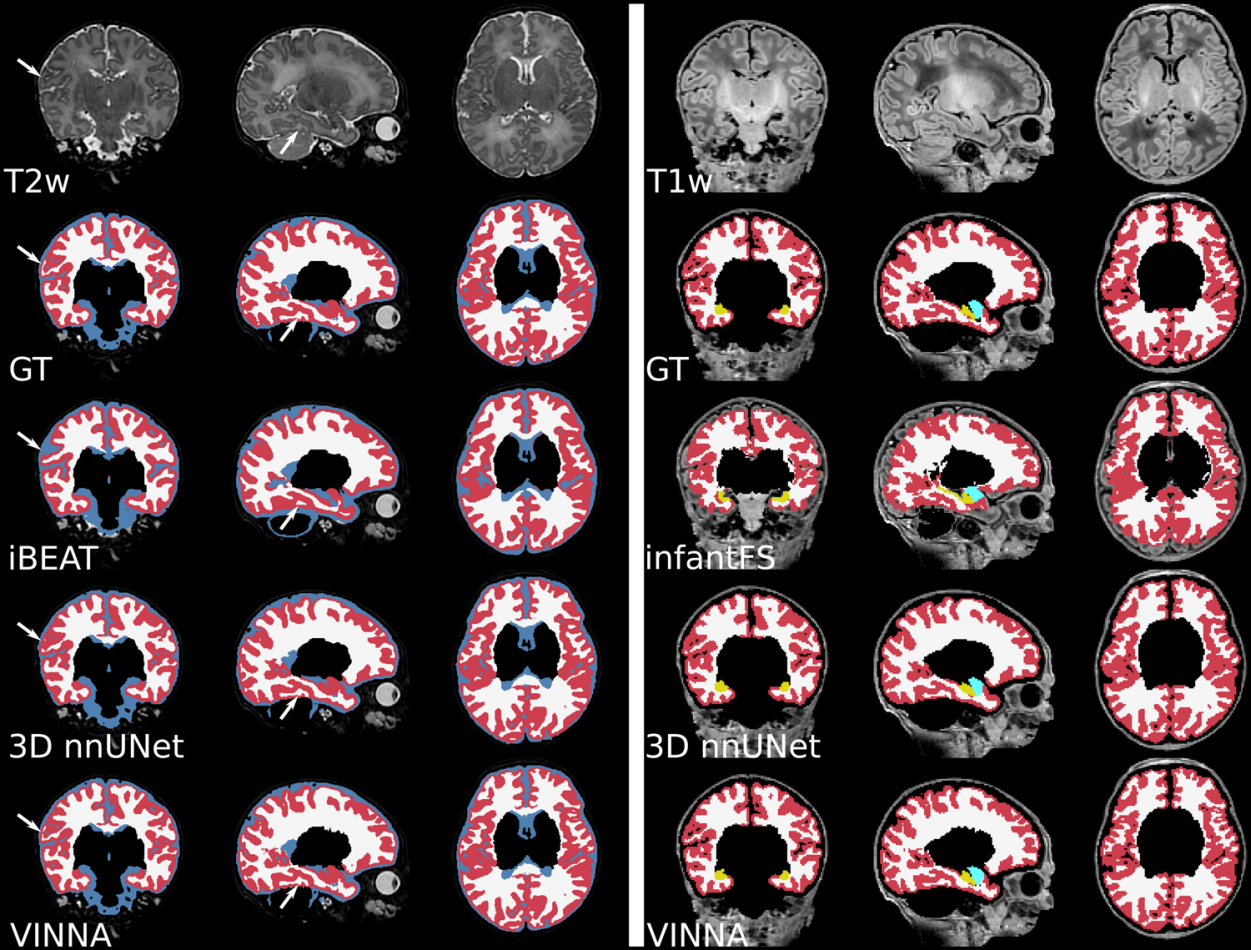
Qualitative T1w and T2w MRI segmentations on a representative scan at 41 weeks. VINNA with the 4-DOF transform module (last row) captures structural details lost in other methods. Comparison of the mapped ground truth (top) and segmentations from iBEAT (third row), 3D nnUNet + exA (fourth row) on a representative participant’s T2w MRI (left). The right side shows the T1w-scan from the same participant at 1.0 mm with ground truth (top), infantFS (third row), and the deep-learning methods.

Overall, the infantFS predictions differ strongly from the mapped dHCP ground truth, specifically for the younger age ranges. The highest similarity is reached for the subcortical structures (amygdala and hippocampus) at 40–46 weeks (DSC of 70.72/70.56 and ASD of 0.7361.23 mm, respectively). The cortex and WM reach a maximum DSC of 60.60/41.62 and ASD of 1.013.20 mm. Qualitative comparison (see Fig. 10, third row, left panel) shows difficulties with the correct location of the GM and WM border on the dHCP T1w MRI. Larger portions of the cortex are under-segmented, and strands of WM are lost compared to the ground truth. The deep-learning methods reach a DSC above 80 and an ASD below 0.5 mm for all structures and age groups. The method closest to the dHCP reference is again VINNA with the 4-DOF transform module (left box), followed by 3D nnUNet + exA (middle box). For a detailed comparison between nnUNet and VINNA on T1w, see Figure 7.

### 3.3 External validation on manual labels (M-CRIB)

To assess generalizability to a different dataset in our target age range (24–44 weeks) and to provide results with respect to a manual reference, we compare the segmentations produced by VINNA, 3D nnUNet + exA, and the dHCP-minimal-processing-pipeline to the 0.62 mm high-resolution T2w scans forming the M-CRIB atlas (Alexander et al., 2019). This dataset contains T2w MRIs from 10 participants and accompanying label maps based on the Desikian-Killiany-Tourville (DKT) atlas (Klein & Tourville, 2012). Note that the labels are not identical to the dHCP-ALBERTs atlas. Hence, we combine the cortical parcels to one label (cortex) for the segmentation comparison and mask all but three structures (WM, hippocampus, and lateral ventricles). As iBEAT does not differentiate between subcortical structures and GM or WM, nor CSF and ventricles, mapping of both, the ground truth and prediction, would be different compared to nnUNet, VINNA, and the dHCP-minimal-processing-pipeline. A fair comparison is therefore only possible between the latter methods, and iBEAT is thus not included in the following section.

**Fig. 11.**
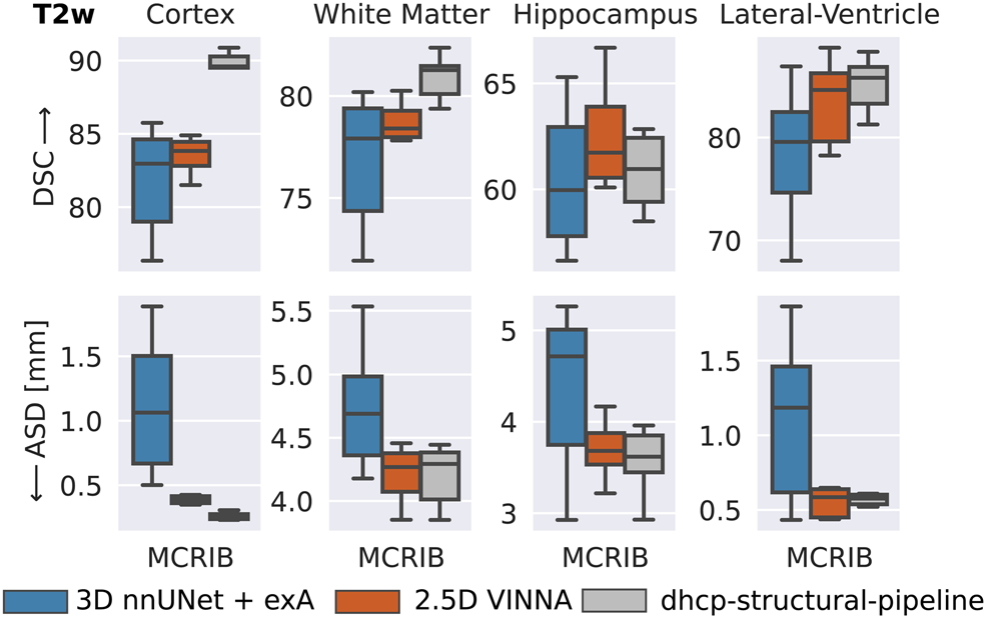
External validation of segmentation performance on the M-CRIB dataset. VINNA (second box) outperforms nnUNet3D (first box) on all four structures and the dHCP-strucutral-pipeline (third box) on the hippocampus. Dice Similarity Coefficient (DSC) and average surface distance (ASD) for cortex, WM, hippocampus, and lateral-ventricles are calculated with respect to harmonized manual labels on 10 subjects from M-CRIB. Note that atlas definition differs between ground truth and predictions.

In Figure 11, the DSC (top) and ASD (bottom) are compared over four structures (from left to right: cortex, WM, hippocampus, and lateral-ventricles) for the deep-learning methods (3D nnUNet + exA and VINNA) as well as the dHCP-minimal-processing-pipeline. Predictions for these methods are all based on the same label definition (dHCP-ALBERTs atlas (Gousias et al., 2012; Makropoulos et al., 2014)). As for the dHCP test set, VINNA outperforms the 3D versions of nnUNet across all four structures with a DSC of 83.83 and an ASD of 0.387 mm on the cortex, 78.41 and 4.268 mm on the WM, 61.73 and 3.682 mm on the hippocampus, and 84.62 and 0.585 mm on the lateral ventricles. Compared to nnUNet, the performance improves on average by 3.36% for the DSC and 19.40% for the ASD. Furthermore, 3D nnUNet + exA incorrectly flips left-right labels for five participants (P02-P04, P08-P09). We restore the lateralization in the presented results as DSC and ASD would have otherwise been close to zero for half of the participants. On the hippocampus, ventricles, and WM performance of VINNA is similar to the dhcp-minimal-processing-pipeline, which is the best performing method. Specifically, predictions of the cortex are closer to the M-CRIB manual labels with the dhcp-pipeline (DSC of 89.60 and ASD of 0.257 mm), which is likely due to the reliance on surface models, while the trained deep-learning models seem to over-segment the cortex (indicated by the similarity of the ASD, but larger differences in the DSC). Note that VINNA performs slightly better on the hippocampus with an increase in DSC by 1.25% and ASD by 0.58%. Due to the low number of participants, significance tests are not applicable for these experiments.

## 4 Discussion

In this paper, we present VINNA—a resolution-independent network for native-resolution neonatal brain MRI segmentation. With VINNA and our novel network-integrated 4-DOF transform module, we address two main difficulties associated with neonate segmentation: resolution non-uniformity across data cohorts and the extended range of head positions in infants.

In contrast to adults, newborn head positioning in the scanner varies significantly due to imaging during sleep, smaller head sizes, and relevant necessary modifications to scanner equipment, such as the padding of head coils. Additionally, while scans are commonly recorded at high resolutions, no uniform standard exists across cohorts. The availability of newborn datasets is also scarcer than that of adult subjects, and the existing collections to date are unlikely to represent that wide diversity in resolutions and head positions.

The current state-of-the-art to address spatial variability such as head positions is data augmentation, which applies randomly sampled scale, rotation, and translation transformations in the native imaging space (i.e., externally to both intensity and label map). In VINNA, we introduce the 4-DOF transform module that can apply such transformations internally as part of the network. As the parameters to the transformation are inputs to the network, they can be randomized during training, similarly to external data augmentation methods. Moving the augmentation operation into the network, so it acts upon feature maps instead of inputs, marks a novel shift for data augmentation strategies. While we only implemented an augmentation of 4 DOFs here, the concept may be generalized to 9 DOFs for 3D or even to warp fields as well as to other tasks such as classification, regression, etc.

We demonstrate that the new network-integrated 4-DOF transform with internal augmentation outperforms state-of-the-art external augmentation approaches in CNN*, VINN, and nnUNet (Isensee et al., 2021) on the dHCP cohort. Across three different resolutions and two modalities, our VINNA achieves the highest DSC (95.33 on average), as well as the lowest ASD (0.102 mm on average). Metric evaluation combined with qualitative inspection indicates that the internal 4-DOF transform module in VINNA better retains high-level details across all resolutions and age groups. It should be noted that VINNA performs augmentation of the feature maps in the first-scale transition. The first (pre-IDB) and last (post-CDB) blocks, therefore, act independent of any interpolation and promote feature detection at an unaltered scale. While the ablative results in Figure 5 indicate better performance than with external augmentation, generalization beyond the scales encountered during training is not assured in these two blocks. Due to their simplicity, the initial, low-level features are, however, often empirically resolution-independent by nature (e.g., Line-Detector).

To better explain the factors and mechanisms driving the performance improvements of VINNA, we review the observation from FastSurferVINN (Henschel et al., 2022) that motivated the extension presented here: in one-to-one comparisons, external augmentation reduces the segmentation performance on sub-millimeter MRIs. While—at first sight—the addition of data augmentation reducing performance seems contradictory, the one-to-one comparison of VINNA and VINNA + exA (see Fig. 5, the only difference is added external augmentation) robustly confirms the observation and extends it from just scaling to rigid transforms.

The positive effect of data augmentation is usually associated with an expansion of the input dataset through equivariant operations. Implementing operations for image and label pairs that are truly equivariant can be difficult. We believe that the loss of information due to image interpolation (lossy interpolation of the label map and image) is larger than previously believed. Internal augmentation, for the first time, offers an alternative approach with interpolation of continuous values in multiple feature maps, reducing the information loss.

Furthermore, the 4-DOF transform module together with the internal augmentation regularizes the latent space of the network, because it imposes an additional constraint: spatial consistency of the feature maps. Compared to equivalent CNN architectures, VINNA (and VINN) also benefit from a reduced capacity requirement to capture a large range of resolutions.

Our comparison to nnUNet highlights that 2D approaches lack contextual information, and fail to provide reliable predictions for whole-brain segmentation. The compromise between mid-range and long-range context in the 2.5D VINNA recovers structural information better and achieves higher segmentation performance across all age groups and structures—even compared to 3D methods. As full-view 3D networks are currently not applicable for high-resolution MRI segmentation due to memory requirements, nnUNet and other 3D networks rely on patch-based processing. In this case, the increased 3D context comes at the cost of limited long-range information and features a smaller field of view, potentially explaining the observed reduction in accuracy compared to 2.5D networks. This finding is in line with previous investigations which found limited performance differences between 2.5D and 3D approaches, even after extensive optimization of the 3D network architectures (Roy et al., 2022).

On the dHCP cohort, VINNA and its 4-DOF transform module also emulates the segmented structures better than traditional state-of-the-art infant pipelines, namely infantFS (Zöllei et al., 2020) and iBEAT (Wang et al., 2023). Notably, infantFS relies on traditional atlas-based segmentation while iBEAT uses a combination of deep-learning and traditional tools with a number of CNNs trained on defined target age ranges. While the re-trained networks (nnUNet + exA and VINNA) reach better results with respect to DSC and ASD, it should be noted that both, infantFS and iBEAT, differ significantly with respect to the returned number and definition of segmented regions. The necessary mapping between the segmentations is bound to introduce a bias, which can not be easily assessed. Additionally, both pipelines cater to a slightly different, larger age range (0–2 years for infantFS and 0–6 years for iBEAT). Consequently, predictions from both methods of the cortex and WM improve for participants closer to the officially supported age range (>40 weeks). Qualitative assessment also shows good performance for the older newborns in iBEAT. infantFS unfortunately fails to correctly capture the cortex and WM on the majority of participants.

The original iBEAT v2.0 paper (Wang et al., 2023) also evaluates performance on the dHCP data and reports a DSC of 0.9 for the WM and 0.85 for the GM. Our results are in concordance with this assessment for the >40 week-old participants (DSC of 0.83 on the GM and 0.88 on the WM). The authors do not provide information on their label harmonization, therefore we cannot infer their reference standard. In contrast to the docker v2.0 version, the cloud version of iBEAT (not available for local data processing) does provide cortical parcellations. Extracting the cortex from the GM label (a combination of both cortical and subcortical GM in the docker version) allows direct comparison to the dHCP solution after merging its cortical structures, possibly explaining performance differences. In summary, iBEAT seems to work well on the supported age range while infantFS is less precise on the dHCP population. Other features included in the pipelines, such as surface generations, are an advantage compared to the proposed VINNA and can help to refine the segmentation of convoluted structures such as the cortex (Fischl, 2012). For our target domain in this paper, however, the VINNA architecture appears to emulate the investigated tissue classes more precisely.

Due to the limited extrapolation capabilities of neural networks, generalizability beyond the training set distribution is, however, uncertain. While the 4-DOF transform module in VINNA serves as a diversification of the training distribution with respect to spatial orientations and image resolution, the base cohort is still only a representation of the dHCP population, that is, all scans encountered during training represent newborns between 24–44 weeks post-menstrual age from a control cohort acquired on the same 3 T Phillips scanner. Therefore, dedicated experimental validation is required to confirm the models’ effectiveness under differing conditions. As for all automated methods, manual quality checks of the predictions are recommended. While VINNA does perform well on M-CRIB, which covers the same age range as the dHCP, generalization to other cohorts is not necessarily guaranteed. Specifically, the T1w image intensities in dHCP appear significantly different from other cohorts which might also explain why infantFS performs poorly on the testing set. The T2w MRIs in dHCP are, on average, of better quality and the dhcp-minimal-processing-pipeline builds the ground truth segmentations based on it (Makropoulos et al., 2018). Additionally, in the early weeks of life, tissue contrast is higher in T2w recordings as the brain is not fully matured and myelination is still ongoing (Dubois et al., 2021; Miller et al., 2012). Structural details and specifically tissue boundaries might be missing, blurred, or ambiguous in T1w MRI. Hence, the imaging data may lack sufficient information to allow correct delineation of the (sub-)cortical structures. This may also explain why the deep-learning networks (i.e., nnUNet, CNN*, VINN), and VINNA are not able to emulate the ground truth on the T1w MRI as closely as on the T2w images. Including a SynthSeg-like intensity augmentation can potentially aid generalization across a wider age range. The method has previously been used in adults to generate contrast-agnostic networks that are able to segment both T1- and T2-weighted images (Billot et al., 2020; Iglesias et al., 2021). Due to the strong contrast changes in the early developmental years, implementing such a generative model may be an interesting direction for future work. However, as mentioned in the introduction, adaptation to the newborn cohort (Shang et al., 2022) showed that the synthetic images still differ considerably from real data leading to performance reduction compared to age-specific models trained on real images. Successful adaptation requires a strategy to close this performance gap.

Better accessibility of newborn datasets would allow diversification of the training sets and subsequently a better representation of the newborn MRI distribution with respect to both, T1w and T2w modalities. It has been shown that an increase in the training corpus alone is extremely effective to boost performance (Henschel et al., 2022; Sun et al., 2017). Age-specific models, such as our VINNA or iBEAT’’s CNNs, are another way to reduce variations and therefore, segregate the problem (i.e., less variations within one age group). However, the limited data availability and non-uniform segmentation labels still pose significant barriers. Models for more specific segmentations than just CSF, WM, and GM are currently not trainable in a supervised fashion due to missing ground truth. In addition, definition of these three structures alone already varies across different atlases and tools, which makes fair method comparisons challenging. Neither manual labels nor automated segmentation tools exist for a unified segmentation definition across different resolutions, modalities, and age ranges. The M-CRIB atlas (Alexander et al., 2019), an infant-specific version of the Desikan-Killiany-Tourville-Atlas (Klein & Tourville, 2012) that is commonly used in adults, provides a first step towards this goal. A consistent structure definition across different stages of life is especially important in the context of longitudinal studies, as segmentation with age-dependent models can induce biases and reduce anatomical consistency (Reuter et al., 2012). How to solve this conundrum is an open question for the future. Several NIH- and internationally-funded initiatives have recently been dedicated to acquire data from newborns (Makropoulos et al., 2018), infants, and pediatric age ranges (Howell et al., 2019; Jernigan et al., 2016; Volkow et al., 2021) as well as adolescence (Karcher & Barch, 2021). Due to the easy integration of varying data resolutions and accommodation for head position variations between infants, toddlers, and adults, our VINNA architecture might prove to be useful in this area once the data and label availability problem is resolved.

Overall, with VINNA, we provide a fast and accurate method for high-resolution subcortical structure segmentation, cortical and WM parcellation of neonatal T1w and T2w MRI which generalizes well across the dHCP cohort. The presented 4-DOF transform module is also easy to integrate into other network architectures and might prove useful in different areas dealing with strong orientation variations. The application to neonates will be made available under VINNA4neonates as an open source package.^8^ Adaptation of the infantFS surface pipeline to the VINNA4neonates predictions, similar to the approach taken in FastSurfer (Henschel et al., 2020) for adults, is an exciting direction for future work.

## Figures and Tables

**Appendix Fig. A1.**
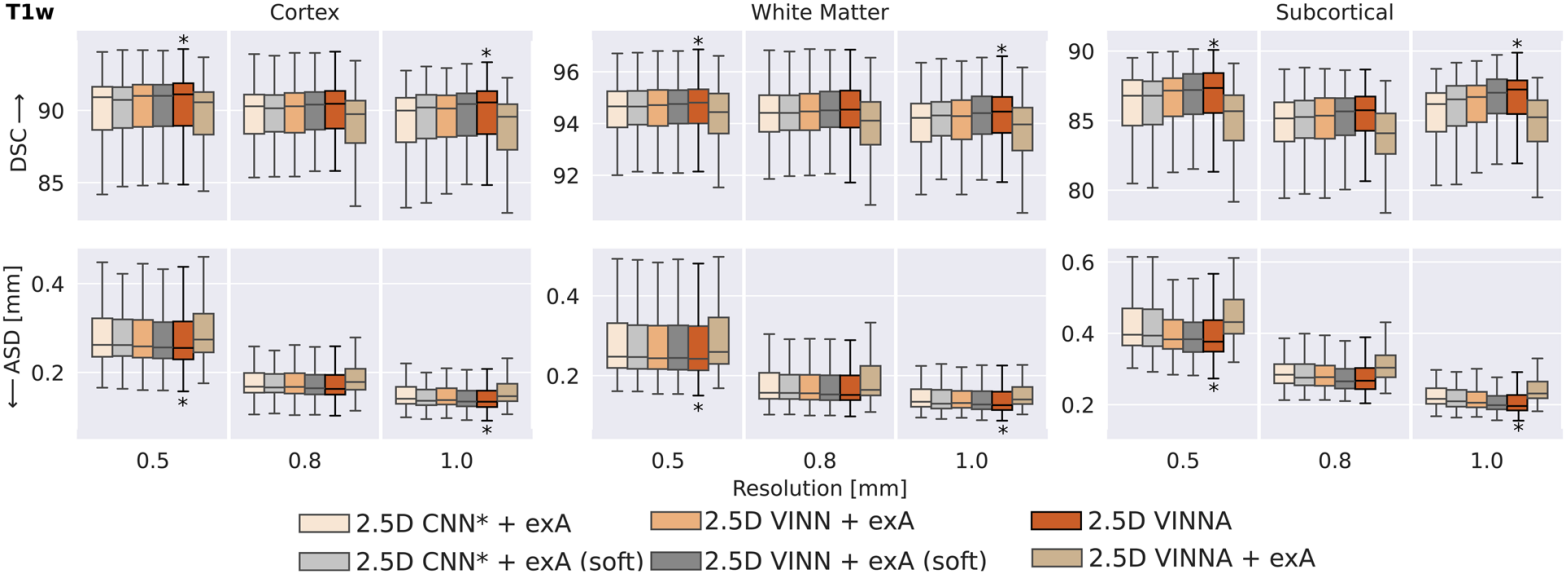
Comparison of approaches for external and internal spatial augmentation on T1w images: Our VINNA method—with the 4-DOF transform module—(fifth box in group) outperforms both state-of-the-art approaches with external Augmentation (CNN* + exA, CNN* + exA (soft), VINN + exA, and VINN + exA (soft); first to fourth box) in Dice Similarity Coefficient (DSC, upper row) and average surface distance (ASD, lower row). This performance advantage is significant on 0.5 mm and 1.0 mm (corrected p < 0.05, indicated with *) and consistent across structures. Combining VINNA with external augmentation (VINNA + exA, last box) reduces performance. exA: external Augmentation. soft: linear interpolation of one-hot encoded feature maps.

**Appendix Table A.**
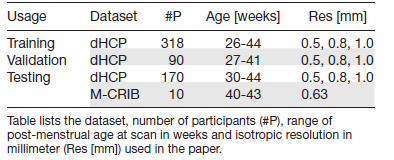
Summary of datasets used for training, validation, and testing.

**Appendix Table B.**
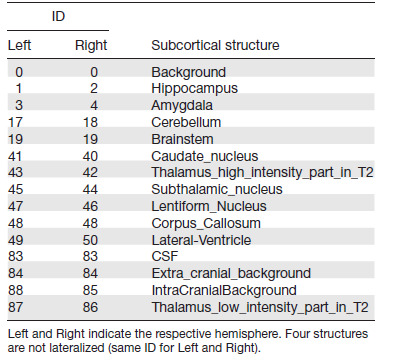
Subcortical segmentations of the dHCP-ALBERTs atlas and matching identifier (ID) equaling the number returned by VINNA for the structure.

**Appendix Table C.**
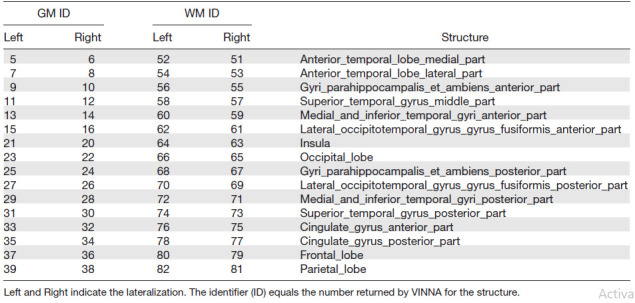
Segmentation parcels of the dHCP-ALBERTs atlas for gray matter (GM) and white matter (WM).

**Appendix Table D.**
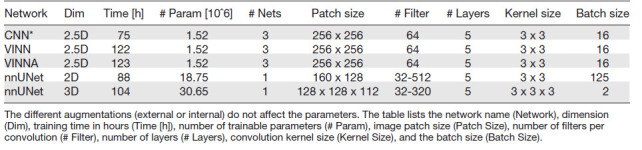
Summary of training parameters for CNN*, VINN, VINNA, nnUNet 2D and nnUNet 3D.

## Data Availability

All MRI datasets used within this article are publicly available, and the open source repositories are cited within the article (Section 2.1). The source code of VINNA4neonates will be made publicly available on Github (https://github.com/deep-mi/VINNA4neonates) upon acceptance.

## Footnotes

1 github.com/Deep-MI/VINNA4neonates upon publication

2
https://github.com/iBEAT-V2/iBEAT-V2.0-Docker

3
https://surfer.nmr.mgh.harvard.edu/fswiki/infantFS

4
https://data.developingconnectome.org/

5
https://ibeat.wildapricot.org/

6
https://github.com/iBEAT-V2/iBEAT-V2.0-Docker

7 To reduce redundancy, we relegate that analysis to the Appendix (Section A.1).

8 on our github pages.

## Appendix

### A.1 Ablation on T1w

Appendix Figure A1 shows DSC (top) and ASD (bottom) for the ablative evaluation external augmentation (exA), optionally with linear interpolation of the one-hot encoded feature maps (exA (soft)) instead of hard label resampling, versus the internal 4-DOF transform modul (VINNA) on the T1w. CNN* + exA (first box) and + exA (soft) (second box) reach the weakest results, followed by VINN + exA (third box) and VINN + exA (soft) (fourth box). Finally VINNA (fifth box) reaches the best result. The performance improves the most on the subcortical structures (2.44% ASD and 0.4% DSC when switching from CNN* to VINN, 5.91% ASD and 0.85% ASD when switching to VINNA). Performance improvements with the 4-DOF transform module in VINNA, is significant for 0.5 mm and 1.0 mm (corrected p < 0.05) and results in an average final DSC of 90.95, 90.03 and 90.36 and ASD of 0.295 mm, 0.2 mm, 0.2 mm for the 0.5 mm. 0.8 mm, and 1.0 mm resolution, respectively. Re-addition of exA to VINNA (sixth box) reduces performance to a similar level as CNN* + exA.

Overall, all models perform better on T2w (Fig. 5) rather than T1w MRIs (Appendix Fig. A1) across all structures. When segmenting a T2w MRI of the same individual, peformance improves for all networks, on average, by 5.7, 3.63 and 5.09 DSC points for the cortex, WM, and subcortical structures, respectively. The ASD difference between T1w and T2w applications nearly doubles for the highest resolution (0.5 mm) with an average of 0.18 mm compared to both lower resolutions (ASD of 0.9 mm for 1.0 and 0.8 mm) for the three structure averages. The DSC point improvement for the T2w images is similar across all resolutions. The trend is consistent for both networks (CNN* and VINN) and augmentation schemes (external versus internal).
